# Are prediction error modifications domain specific in autism but domain general in ADHD?

**DOI:** 10.1162/IMAG.a.942

**Published:** 2025-10-17

**Authors:** Irene S. Plank, Anna Yurova, Alexandra Pior, Julia Nowak, Afton M. Bierlich, Boris Papazov, Zhuanghua Shi, Christine M. Falter-Wagner

**Affiliations:** Department of Psychiatry and Psychotherapy, LMU University Hospital, LMU Munich, Munich, Germany; Department of Sociology, Faculty of Behavioral and Social Sciences, University of Groningen, Groningen, Netherlands; Department of Psychology, Faculty of Psychology and Neuroscience, Maastricht University, Maastricht, Netherlands; Department of Radiology, LMU University Hospital, LMU Munich, Munich, Germany; NeuroImaging Core Unit Munich (NICUM), LMU Munich, Munich, Germany; Department of Psychology, LMU Munich, Munich, Germany

**Keywords:** prediction error, Bayesian Brain, predictive coding, autism, ADHD, face processing, emotion processing

## Abstract

In the last decade, several theories have proposed explanations of autism spectrum disorder (ASD) based on the Bayesian Brain hypothesis. Research on repetition suppression suggests that neural correlates of prediction errors in ASD might be domain specific with larger differences for faces than for objects. Contrastingly, research assessing mismatch negativity in attention-deficit/hyperactivity disorder (ADHD) indicates domain-generally modified prediction errors. Therefore, we captured neural correlates of prediction errors to colours and emotions in adults with ADHD or ASD to assess domain specificity of prediction error modifications. We used a multi-feature roving paradigm where we assessed predictive processes regarding unattended, task-irrelevant faces. We extracted task specific precision-weighted prediction errors and prediction strength for emotions and colours of the faces separately using a generative model, specifically a Hierarchical Gaussian Filter. While we found neural correlates of colour precision-weighted prediction errors and prediction strength as well as emotion prediction strength in the pooled sample regardless of group, we did not find any differences in neural correlates of emotion or colour precision-weighted prediction errors or prediction strength between our groups. These results suggest preserved neural correlates of precision-weighted prediction errors in ASD and ADHD for low-level visual features and potentially complex social information.

## 1 Introduction

Interacting with our environment requires us to constantly interpret sensory inputs that we receive and balance new information with existing expectations. To achieve smooth interactions, this process needs to happen fast. Thus, our brain makes predictions about what is going to happen next: this constant predictive process speeds up processing and allows us to prepare reactions earlier. The importance of prediction has been formalised in the Bayesian Brain framework (Clark, 2013; K. Friston, 2018). According to this framework, the brain makes predictions based on priors which are internal models about the world. These priors are combined with sensory experiences to create our perception of the world. A process of continuous optimisation of perception is enabled by adjustments of precision (i.e., inverse variance). Through this optimisation process, prediction errors (i.e., discrepancy between priors and sensory input) are supposed to be reduced as much as possible. These processes of prediction and prediction errors happen on multiple levels. For instance, a common computational model based on the Bayesian Brain framework, the Hierarchical Gaussian Filter (HGF, Mathys et al., 2011, 2014), models the development of belief states as belief state trajectories on three levels: the first level regards the belief about the stimulus occurrence, the second level models the belief about the stimulus probability, and the third level models environmental volatility. Neural correlates of hierarchical prediction errors have been researched extensively in adults (Iglesias et al., 2013; Stefanics et al., 2019; Weilnhammer et al., 2018). A recent meta-analysis revealed evidence for domain specificity of these error signals with midbrain prediction error signals being associated with cognitive and reward learning tasks, while insula prediction error signals were associated with a wide range including perceptual, cognitive, and social prediction errors (Corlett et al., 2022).

In the last years, several theories have postulated predictive impairments to underlie autism spectrum disorder (ASD), many of which were based on the Bayesian Brain framework (Brock, 2012; for a review see Cannon et al., 2021; K. J. Friston et al., 2013; Lawson et al., 2014; Pellicano & Burr, 2012; Sinha et al., 2014; van de Cruys et al., 2014). A recent study demonstrated differences in neural correlates of prediction errors in autistic compared with non-autistic adults (Sapey-Triomphe et al., 2023). Specifically, the authors used a task in which participants had to learn associations between tone cues and an upcoming perceptual decision with the tone predicting a rotation direction of a dot with 75% probability. This probability with which a tone predicted the following decision could suddenly change, with a tone previously predicting a left rotation with 75% now predicting a left rotation with only 25% probability. While there was a large overlap in areas associated with prediction errors between autistic and non-autistic adults, the authors also found associations between neural activation and prediction errors to be decreased in autistic adults in the anterior cingulate cortex and the putamen. These results suggest that predictive impairments postulated for ASD are associated with differences in neural correlates of prediction errors.

While no research so far has investigated the domain specificity of prediction error modifications in ASD, research focusing on neural adaptation could indicate a stronger difference between autistic and non-autistic adults for social sensory input. Ewbank et al. (2017) investigated repetition suppression for task-irrelevant faces and objects, resulting in decreased neural activation with repeated presentation of the same feature, for example, face identity or object shape. They found that autistic adults showed less repetition suppression for faces but not for objects. D’Mello et al. (2023) replicated this pattern: autistic adults had reduced repetition suppression for faces along with unchanged repetition suppression for objects, printed or spoken words. Tam et al. (2017) found only non-autistic adults exhibited repetition suppression in the amygdala for faces. Furthermore, Kleinhans et al. (2009) observed weaker amygdala repetition suppression to neutral faces in autistic adults, although fusiform repetition suppression matched that of non-autistic adults. Utzerath et al. (2018) showed equally strong repetition suppression in autistic and non-autistic adolescents, but autistic and non-autistic adolescents diverged in their neural activation between expected and unexpected stimuli. However, not all studies found decreased repetition suppression to faces in ASD (Webb et al., 2010). Multiple studies report reduced repetition suppression in ASD for other types of stimuli, including human and non-human sound (Font-Alaminos et al., 2020; Kolesnik et al., 2019; Millin et al., 2018; Ruiz-Martínez et al., 2020), tactile stimuli (Piccardi et al., 2021), and dot patterns during an implicit prototype learning task (Schipul & Just, 2016). Taken together, these results suggest that neural correlates of prediction errors are decreased in ASD and that this decrease could be domain specific for images, only applying to social but not non-social features.

While less research has focused on prediction errors in people with attention-deficit/hyperactivity disorder (ADHD), there are several indications for possible modifications of neural correlates of prediction errors in ADHD. First, both auditory and visual oddball paradigms revealed decreased mismatch negativity amplitudes in adults with ADHD compared with adults without ADHD (Dang et al., 2024; Hsieh et al., 2021; Kim et al., 2021), which has been conceptualised as an indicator of prediction errors (Cheng et al., 2016; Gonzalez-Gadea et al., 2015). Indeed, Gonzalez-Gadea et al. (2015) found a reduced P300 amplitude for unexpected deviant stimuli in children with ADHD which could reflect weaker top-down expectations resulting from an overemphasis on novel sensory evidence. Second, neurobiological theories have emphasised decreases or imbalances in dopamine and noradrenaline levels as underlying impairments in ADHD (Véronneau-Veilleux et al., 2022; Ziegler et al., 2016). Both neurotransmitters seem to play a vital role in processing prediction errors (Diederen & Fletcher, 2021). Specifically, dopamine was proposed to modulate the precision weighting of prediction errors, encoding the reliability of a prediction error (Diederen & Fletcher, 2021). While, in contrast to ASD research, no theoretical model of ADHD focuses on predictive coding explicitly (Marais & Roche-Labarbe, 2025), Ziegler et al. (2016) deduced hypotheses from common neurobiological theories regarding possible increases and decreases in reward prediction errors. Reward prediction errors are caused by differences between expected and received rewards and can be positive or negative, depending on whether the reward exceeds or fails to meet expectations, respectively (Schultz, 2017). Ziegler et al. (2016) propose that the Basal ganglia model (Frank et al., 2007) predicts decreased positive but increased negative reward prediction errors, while the dynamic developmental theory (Sagvolden et al., 2005) predicts that both positive and negative reward prediction errors are decreased. Indeed, adolescents with ADHD showed reduced reward prediction error processing in the medial prefrontal cortex during a reversal learning paradigm compared with adolescents without ADHD (Hauser et al., 2014), but the study did not separate positive from negative reward prediction errors. While research on prediction errors beyond reward learning remains limited, imbalanced levels of dopamine and noradrenaline in ADHD could underlie a broad, domain-general predictive impairment in ADHD, contrasting with the more domain-specific patterns in ASD.

In this preregistered study, we aimed to assess domain specificity of prediction-error processing in ASD and ADHD using a multi-feature roving paradigm (Stefanics et al., 2019). Assessing possible differences in domain specificity could sharpen our views on underlying mechanisms: if both groups show similar differences in prediction, this might indicate a shared mechanism; but if ASD reveals domain-specific differences, that split would indicate differentiated mechanisms and foster our understanding of how different predictive processing mechanisms might be related to different sets of neurocognitive profiles. We used two Hierarchical Gaussian Filters (HGF, Mathys et al., 2011, 2014) to model prediction strength (PS) and precision-weighted prediction error (pwPE) of repeated presentations of colour and emotion in faces separately. Extracted belief state trajectories were used as parametric modulators of neural activation to compare neural correlates of pwPE and PS for low-level visual features, such as colour, and complex, social information, such as emotions. We hypothesised differences in neural correlates of pwPE between both experimental groups and the comparison group, and we explored whether any observed differences are specific to emotion pwPE or domain general for both emotion and colour pwPE. Furthermore, we expected to replicate neural correlates of pwPE and PS as reported before in adults without psychiatric diagnoses (Stefanics et al., 2019), both in our comparison group and in the pooled sample of all groups. Finally, we complemented our HGF-based model with a conventional model capturing repetition suppression (RS) to colours and emotions, for which we hypothesised reduced RS for emotions in autistic adults compared with adults without psychiatric diagnoses.

## 2 Method

This study was part of a larger project, which was approved by the ethics committee of the Medical Faculty of the LMU (reference: 22-0561). The study was preregistered on the Open Science Framework (OSF): https://osf.io/8szc7. The hypothesis regarding neural correlates of PS and pwPE in the pooled sample was added after data collection was concluded but before analyses were conducted. All other hypotheses were preregistered before data collection. We did not assess the hypotheses regarding face processing as the short interstimulus interval in combination with the high pass filter would significantly decrease power. Preprocessing and analysis of eye-tracking and behavioural data were performed using R *4.4.1* (R Core Team, 2021) in RStudio (RStudio Team, 2020). Neuroimaging data were preprocessed using fMRIPrep *23.0.2* (Esteban et al., 2019) and analysed using FSL *6.0.7.10* (Jenkinson et al., 2012; Smith et al., 2004). The experiment was presented using NBS Presentation® *23*. We share all code as well as preprocessed, anonymised data on GitHub: https://github.com/IreneSophia/EMBA_VMM. Variances are reported as standard errors, and coordinates are in MNI space throughout the manuscript.

### 2.1 Participants

We recruited 88 adults (25 with ADHD, 32 with ASD, 31 in the comparison group of which 7 were invited to pilot the task) for this study by posting flyers online, at the University Hospital of the Ludwig-Maximilians-Universität München (LMU) Munich and in psychotherapist practices. Some participants were directly contacted through an internal participant database. Participants received either study credits or monetary compensation for their time as well as compensation for travel expenses, if relevant. Study credits are required by psychology courses and obtained by taking part in research studies at the university. Due to the high volume of interested young women for the comparison group, we stopped recruitment in this group early to achieve a balanced sample. We asked 26 of our autistic participants whether they prefer identity-first or person-first language, which revealed balanced preferences (identity-first: 42.31%, person-first: 38.46%, rest no preference). Therefore, we use both types of terms throughout this manuscript.

All participants had no neurological diagnosis, sufficient knowledge of the German language to understand the study instructions, an estimated IQ of at least 70, unimpaired or corrected-to-normal vision, were between 16 and 45 years old, and suitable for scanning with magnetic resonance imaging (MRI). Participants in the comparison group had no psychiatric diagnoses and took no psychotropic medication, while participants in the clinical groups had either a formal diagnosis of ASD or ADHD but never both. We validated existing formal diagnoses by viewing medical reports. Furthermore, the diagnoses of most of our autistic participants were verified by a trained clinician in the outpatient clinic for autism at the Department of Psychiatry. Participants and possible guardians provided written and informed consent. Additionally, participants were excluded in the following cases: acute suicidality (one autistic participant), self- or other-harming behaviour (no participants), excessive motion in the MRI scanner (one autistic participant; defined as mean framewise displacement above 0.55 mm or maximum framewise displacement above 5 mm based on Jenkinson et al., 2002), low accuracy in the change detection task as explained in detail below (two comparison and three autistic participants), abnormal findings in the neuroradiological assessment (one participant with ADHD), and change in fulfilment of inclusion criteria (one participant). Furthermore, data from four participants could not be used due to technical difficulties (three participants with ASD and one with ADHD), and one participant with ADHD asked to stop the scanning session prematurely.

We continually preprocessed data with the goal of analysing 22 participants per group. This goal was based on a power analysis to achieve at least 80% power for each of the tasks the participants performed as part of the larger project. For this study, a difference between two of the three groups achieved a power of 95.7% based on PANGEA (for details, see the preregistration, Westfall, 2016). The final sample included neuroimaging and behavioural data from 67 participants, 23 adults with ADHD, 22 adults with ASD, and 22 adults without any psychiatric diagnoses (comparison group, COMP; see Table 1). Approximately half of the participants with ADHD were taking ADHD medication at the time of the study, one with atomoxetine, four with amphetamines (one of which took additional antidepressants), eight with methylphenidate (two of which took additional antidepressants), and two with antidepressants only. Furthermore, 10 of the 22 autistic participants took either antidepressants and/or antipsychotics and 1 took an antiepileptic. Bayesian contingency tables revealed strong evidence against differences in gender composition as well as moderate evidence against differences in education level between groups (gender: log(*BF_10_*) = -2.932; education: log(*BF_10_*) = -1.835). Bayesian ANOVAs of ranked age and estimated IQ revealed moderate evidence against group differences regarding age (log(*BF_10_*) = -1.786) as well as estimated IQ (log(*BF_10_*) = -1.101). The sample for which eye-tracking data were analysed was slightly smaller than the sample used to test our hypotheses. The eye-tracking sample consisted of 42 participants overall (16 with ADHD, 11 with ASD, and 15 in the comparison group).

**Table 1.** Group comparisons on socio-demographic variables and clinical self-assessment.

Measurement	ADHD	ASD	COMP	log(BF)
ASRS-v1.1	44.30 ± 2.45 (16 to 62)	30.36 ± 1.85 (14 to 52)	24.95 ± 1.76 (13 to 42)	10.858*
Age	26.96 ± 1.51 (17 to 43)	29.36 ± 1.69 (20 to 45)	27.77 ± 1.22 (22 to 42)	-1.786
BDI	7.78 ± 1.57 (0 to 33)	7.82 ± 1.23 (0 to 21)	2.45 ± 0.66 (0 to 13)	4.808*
Education1	3.57 ± 0.23 (1 to 5)	3.91 ± 0.21 (2 to 5)	4.36 ± 0.18 (2 to 5)	-1.835
Gender2	9 – 12 – 2	14 – 8 – 0	10 – 12 – 0	-2.932
IQ estimate	106.65 ± 2.23 (84 to 123)	114.98 ± 2.98 (96 to 144)	109.70 ± 2.10 (92 to 131)	-1.101
RADS-R	94.09 ± 8.15 (18 to 148)	148.09 ± 8.80 (55 to 217)	44.50 ± 7.62 (9 to 142)	20.882*

Means and standard errors are shown for continuous variable and counts for gender distribution. Bayes Factors (BF) were computed using Bayesian ANOVAs except for education and gender distribution where contingency tables were used. Log-transformed BFs above 3 are considered credible evidence in favour of a difference.

*Note*. ADHD = adult attention-deficit/hyperactivity disorder; ASD = autism spectrum disorder; ASRS = Adult ADHD Self-Report Scale; COMP = comparison group of adults without psychiatric diagnoses; IQ = estimated intelligence quotient from verbal and non-verbal IQ; RADS-R = Ritvo Autism Diagnostic Scale—Revised. ^1^Education: ranging from 1 = “no educational or vocational training” to 5 = “university degree”; ^2^gender categories: female—male—gender-diverse/agender/nonbinary; asterisks highlight credible differences. Asterisks indicate at least moderate evidence in favour of group differences as assessed by a Bayesian ANOVA.

### 2.2 Procedure

All testing was conducted at the NeuroImaging Core Unit Munich (NICUM), LMU Munich (https://www.en.nicum.uni-muenchen.de/index.html). After their arrival at the NICUM, all participants were informed about the study procedure and had the opportunity to ask questions. Informed consent was obtained from all participants for being included in the study. Next, participants filled out questionnaires and intelligence assessments to ensure all inclusion criteria were fit. Specifically, this included a sociodemographic questionnaire, the Beck Depression Inventory (BDI, Hautzinger et al., 1994), as well as verbal (MWT-B, Lehrl, 2005) and nonverbal (CFT-20R, Weiß, 2006) intelligence assessments, which we averaged for a general IQ estimate. Additionally, they filled out the Ritvo Autism Diagnostic Scale—Revised (RADS-R, Rausch et al., 2024) and the Adult ADHD Self-Report Scale (ASRS-v1.1, Kessler et al., 2005). The RADS-R and the ASRS-v1.1 were used as clinical self-assessments of symptom severity for the ASD and ADHD participants. All questionnaires were filled out on an iPad using CentraXX (KAIROS GmbH), while pen and paper were used for intelligence assessments. Furthermore, participants performed behavioural tasks assessing facial emotion recognition which was comparably impaired in the ADHD and the ASD groups (Plank et al., 2024), face attention bias which was increased in the ADHD group (Plank, Nowak et al., 2025), and probabilistic associative learning which was spared in both ASD and ADHD (Plank, Pior et al., 2025). The order of the behavioural tasks was counterbalanced. After finishing the behavioural tasks, we conducted the neuroimaging session where we collected structural and functional data from the participants. Last, the Toronto Alexithymia Scale (TAS-20, Popp et al., 2008) was filled out at the end of the testing. Participants were offered breaks in between tasks as well as between the behavioural and neuroimaging sessions. One participant conducted the fMRI session on a different day due to a piercing that had to be removed by a specialist. Most testing sessions lasted approximately 3 to 4 hours, of which about 1 hour was spent in the MRI scanner.

### 2.3 Visual mismatch and change detection task

**Fig. 1.**
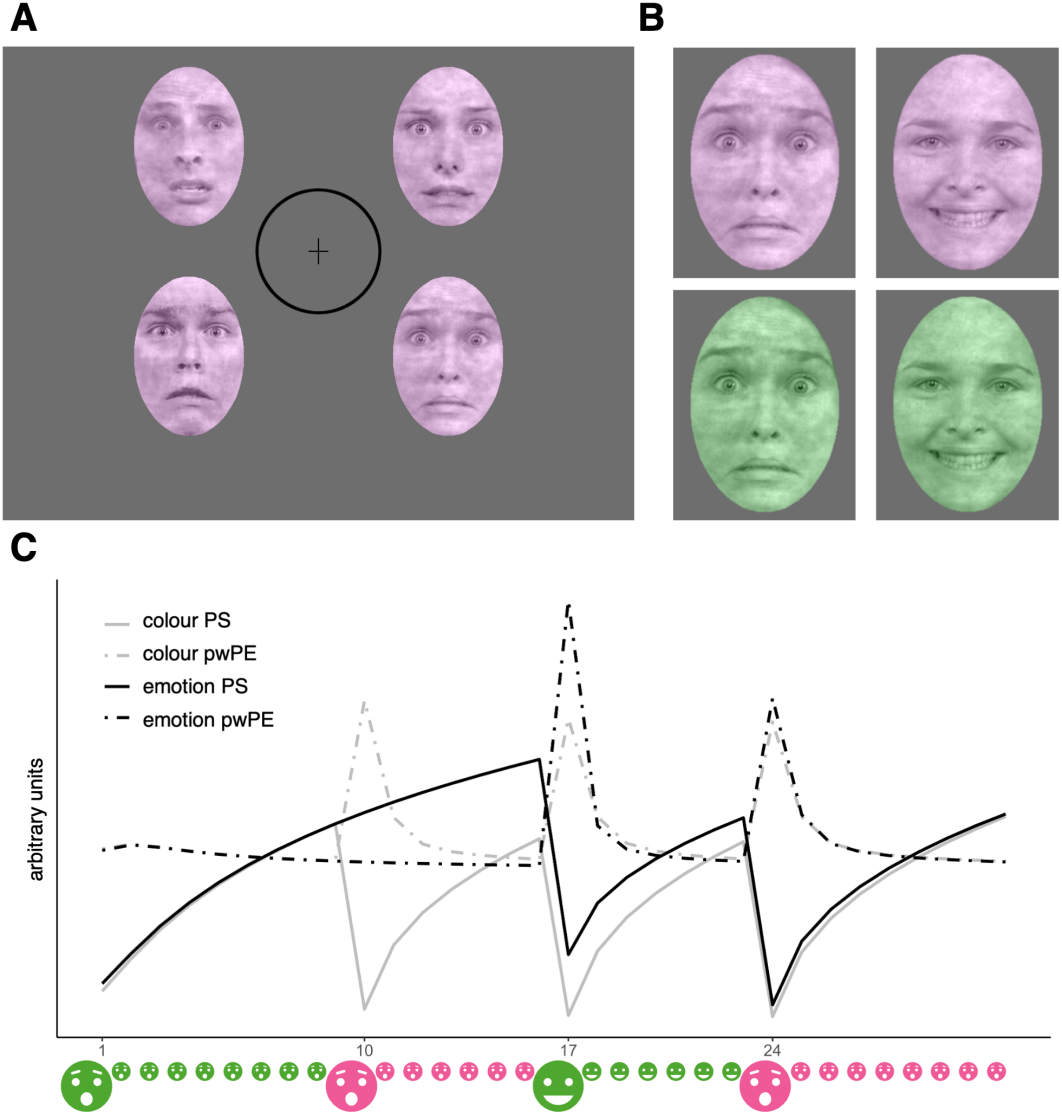
Task design. (A) The presentation of the faces in the periphery as well as the fixation cross in the centre of the screen; changes of which were supposed to be detected by the participants. The circle shows the area defined as centre for the analysis of dwell times—this circle was not presented to participants. (B) The same facial identity in all four conditions—fearful pink, happy pink, fearful green, and happy green. (C) The development of PS (solid lines) and pwPE (dashed lines) concerning emotions in black and colours in grey. The x-axis shows trials represented by an emoji capturing the colour and emotion of the presented tetrad. Over the course of a block, predictions increase in strength. With the start of a new block, either the colour, the emotion, or both stimulus features change, resulting in corresponding pwPE; for instance, colour changes for the second time and emotion for the first time on trial 17. Both changes lead to a pwPE, however, the greater PS regarding emotions results in a bigger pwPE.

Participants completed a multi-feature roving visual mismatch paradigm in the MRI scanner where their task was unrelated to the stimuli of interest. The task was programmed based on Stefanics et al. (2019) in NBS Presentation®, using the same coloured stimuli and the same stimulus orders which were kindly provided by the authors. Stefanics et al. (2019) took the faces from the Radboud Faces Database (Langner et al., 2010) and processed them with the SHINE toolbox (Willenbockel et al., 2010). Specifically, participants were asked to press a button whenever they detected a change in the fixation cross. The fixation cross randomly switched from long to wide and back (on average eight times per minute). While participants attended the fixation cross, tetrads of faces were additionally presented for 200 ms followed by 550 ms of the fixation cross on its own. Each face in a tetrad portrayed the same emotion (fear or happiness) and was coloured in the same colour (pink or green, see Fig. 1). Blocks of five to nine consecutive tetrads were coloured in the same colour and portrayed the same emotion, building up predictions regarding the colour and emotion of the next tetrad. Between blocks, the emotion, the colour, or both changed to elicit a mismatch reaction in the form of a pwPE. Participants were presented with 2 runs of 1190 trials each, with a short break of 12 seconds in the middle of each run. All participants performed the same 4 stimulus sequences of 595 trials each; the order of those 4 sequences was counterbalanced across participants. Each run took about 15 minutes, resulting in 30 minutes of fMRI task duration. Only participants whose data of both runs could be used were included in the analysis.

### 2.4 Modelling of prediction strength and precision-weighted prediction errors

We used the standard HGF as published in the TAPAS toolbox version 6.0.1 (Frässle et al., 2021) to model belief state trajectories about emotions and colours separately, similar to Stefanics et al. (2019). The HGF is a generative model of perceptual inference, allowing one to infer hierarchically related hidden environmental states that generate sensory inputs (Mathys et al., 2011, 2014). Since the environmental volatility was stable in our task, we adjusted the configuration of the HGF to decouple the second and third levels by setting both the contribution of the third level to the second level and the variance of the third level parameters to zero. PS is captured by the posterior mean of the belief on the second level (μ_2_), while pwPE at the second level (ε_2_) captures the update of the PS at the second level and can be computed as the prediction error at the first level divided by the inverse observation noise, or posterior precision, at the second level (Mathys et al., 2014).

### 2.5 Data collection in the scanner

Neuroimaging data were collected using a 3T MAGNETOM Prisma MRI scanner (Siemens Healthineers) with a 20-channel head array coil. First, T1-weighted magnetisation-prepared rapid gradient echo images were acquired (176 slices, 1 mm^3^ resolution, TR = 2250 ms, TE = 2.15 ms, FA = 8°, FOV = 256 mm), followed by functional imaging using echo-planar imaging during the visual mismatch task (2 runs, 38 slices, 3 mm^2^ resolution, TR = 2451 ms, TE = 30 ms, FA = 77°, FOV = 210 mm). Functional data from pilot participants as well as 1 participant who was included in the analysis were gathered in 394 volumes per run, while all other functional data were collected in 380 volumes per run. Eye movements of the right eye were tracked with 500 Hz using an EyeLink 1000 Plus eye tracker (SR Research) during each functional run except for participants for which calibration was not possible, for example, due to thick glasses. Before each functional run, spin echo field maps were collected (38 slices, 3 mm thickness, TR = 8000 ms, TE = 66 ms, FA = 90°, FOV =210 mm). Last, we obtained fluid-attenuated inversion recovery (FLAIR) images for a neuroradiological assessment (176 slices, 1 mm^3^ resolution, TR = 5000 ms, TE = 388 ms, FOV = 256 mm).

### 2.6 Preprocessing of the fMRI data

We conducted fMRI pre-processing using the automated fMRIPrep *23.0.2* pipeline (Esteban et al., 2019), following NIFTI conversion with dcm2bids *2.1.7* (Boré et al., 2023). The detailed pre-processing pipeline generated by fMRIPrep is provided in the Supplementary Materials S4. T1-weighted anatomical images underwent a series of pre-processing steps, including non-uniformity correction, skull stripping, brain tissue segmentation, surface reconstruction using FreeSurfer *7.3.2* (Dale et al., 1999), spatial normalisation, and registration to the MNI152 template. Each functional scan underwent field map correction, slice time correction, and co-registration to the corresponding T1-weighted image. Last, spatial smoothing (6 mm FWHM Gaussian kernel) and motion artefact removal using ICA-AROMA (Pruim et al., 2015) were applied. The brain mask extracted from FreeSurfer was used to mask the preprocessed functional scans.

### 2.7 Analyses of the fMRI data

As preregistered, we created two general linear models per run per participant on the subject level. Both models were realised using FSL FEAT (Smith et al., 2004), all predictors were convolved with the double-gamma haemodynamic response function and none of them was orthogonalised (Mumford et al., 2015). Both models included the following predictors for each tetrad onset with a duration of 0: intercept, colour (-1 for pink, 1 for green), and emotion (-1 for happy, 1 for fearful). The model focusing on neural adaptation additionally included parametric modulators capturing the number of consecutive tetrads showing the same colour and emotion combination as well as the number of consecutive tetrads showing the same colour or emotion separately. The second model focusing on the HGF belief state trajectories additionally included absolute and scaled PS and pwPE, both for colours and emotions separately, and was chosen to closely match the model used by Stefanics et al. (2019). Last, both models included a predictor modelling participant-specific button presses as well as six motion regressors (three translational, three rotational) extracted from fMRIPrep as confounds. The two runs were then averaged within subject.

*T*-tests were used to evaluate hypotheses on the group level: one-sample *t*-tests to assess neural correlates of neural adaptation, PS, and pwPE, as well as unpaired *t*-tests to assess differences between the groups. Staying in line with Stefanics et al. (2019), we did not preregister the inclusion of any subject-specific covariates in these models. However, to ensure that our results were not influenced by subtle differences in gender distribution or age, we ran explorative comparisons with gender and age added as covariates. For the directed hypothesis concerning neural adaptation as well as the two-sample *t*-tests, *t*-contrasts were used, while we used *f*-contrasts for the undirected hypotheses concerning PS and pwPE. All hypothesis-guided and explorative tests were evaluated using threshold-free cluster enhancement (Smith & Nichols, 2009) and family-wise error correction (*α* = 0.05) as implemented in FSL, with the analyses being restricted to the preregistered ROIs: areas associated with face processing for colour pwPE (bilateral fusiform gyri, Kanwisher et al., 1997) as well as areas associated with face and emotion processing for RS and emotion pwPE (bilateral anterior cingulate cortices, amygdalae, insulae, and precunei, as well as right supramarginal gyrus and posterior temporal gyrus, Plank et al., 2022). ROIs were defined based on the toolbox AAL3v2 (Rolls et al., 2015, 2020; Tzourio-Mazoyer et al., 2002) and combined to a single mask containing all ROIs for each specific hypothesis. Furthermore, we computed Bayes Factors using a Bayesian ANOVA based on the extracted activation from the significant clusters in the pooled sample for each of the parameters and corrected for multiple comparisons following Westfall et al. (1997) as described in de Jong (2019). Last, we explored differences between groups without preregistered hypotheses as well as neural correlates outside of the ROIs in additional whole-brain analyses.

### 2.8 Preprocessing and analyses of the behavioural and eye tracking data

In total, each participant was presented with 240 fixation cross changes across both runs. Button presses within 2 seconds following a fixation cross change were considered hits. Other button presses were considered false alarms. Only participants with fewer than 40 misses and 40 false alarms in each run were included in the analysis of both behavioural and neuroimaging data. Discrimination rate was calculated as the difference between hits and false alarms. Bayesian linear models as implemented in brms (Bürkner, 2017) were used to assess possible differences between groups regarding hit reaction times and discrimination rate. We set the priors based on the literature and data-naive modelling of the prior predictive distribution. All models were evaluated using simulation-based calibration (Modrák et al., 2023; Schad et al., 2020), and all contrasts were set to sum coding. Models with divergence issues or poor posterior predictive fit were adjusted before inferences were drawn using the brms::hypothesis function. The model of the reaction times was based on a shifted lognormal distribution and only included hits. Due to divergence issues, reaction times were aggregated using the median and modelled with the predictor group. To model discrimination rate using a Poisson distribution, negative discrimination rate was calculated by subtracting the discrimination rate of each participant from the perfect discrimination rate. The model included the predictor group and group-level intercepts for the participants.

We used fixations as automatically extracted by the EyeLink 1000 Plus eye tracker (SR Research) to assess fixation duration to the fixation cross in the centre as instructed and not the tetrads of faces in the periphery. Only participants with an average calibration error below 1° for both runs were included in the analysis (n = 42). For each participant, we calculated centre dwell time as the percentage of fixation duration to the centre from the total fixation duration. The centre was defined as a circle area of interest with a radius of 100 pixels. The data were rank transformed to achieve a normal distribution, and group differences were assessed using a Bayesian ANOVA as implemented in the BayesFactor package (Morey et al., 2022).

## 3 Results

### 3.1 Clinical self-assessment of symptom severity

ADHD participants scored higher on the ASRS-v1.1 than both ASD and COMP participants (ADHD vs. ASD: log(*BF*) = 5.99; ADHD vs. COMP: log(*BF*) = 11.472), while there was no difference between ASD and COMP participants (ASD vs. COMP: log(*BF*) = 0.550). Regarding the RADS-R, both clinical groups scored higher than the COMP participants (ADHD vs. COMP: log(*BF*) = 5.79; ASD vs. COMP: log(*BF*) = 19.036), and ASD participants scored higher than ADHD participants (ADHD vs. ASD: log(*BF*) = 5.989). Furthermore, ASD and ADHD participants reported higher depressive symptoms as measured by the BDI than COMP participants with ASD and ADHD participants not differing from each other (ASD vs. COMP: log(*BF*) = 4.201; ADHD vs. COMP: log(*BF*) = 2.376; ADHD vs. ASD: log(*BF*) = -1.221). Only 4 of the 22 COMP participants scored above the cut-offs on these questionnaires, 1 on the RADS-R and 3 on the ASRS-v1.1. However, the majority of ADHD participants scored above the cut-off of the RADS-R, specifically 56.5%, while only 22.7% of the ASD participants scored above the cut-off for the ASRS-v1.1.

### 3.2 Neural correlates of prediction strength and precision-weighted prediction error

**Fig. 2.**
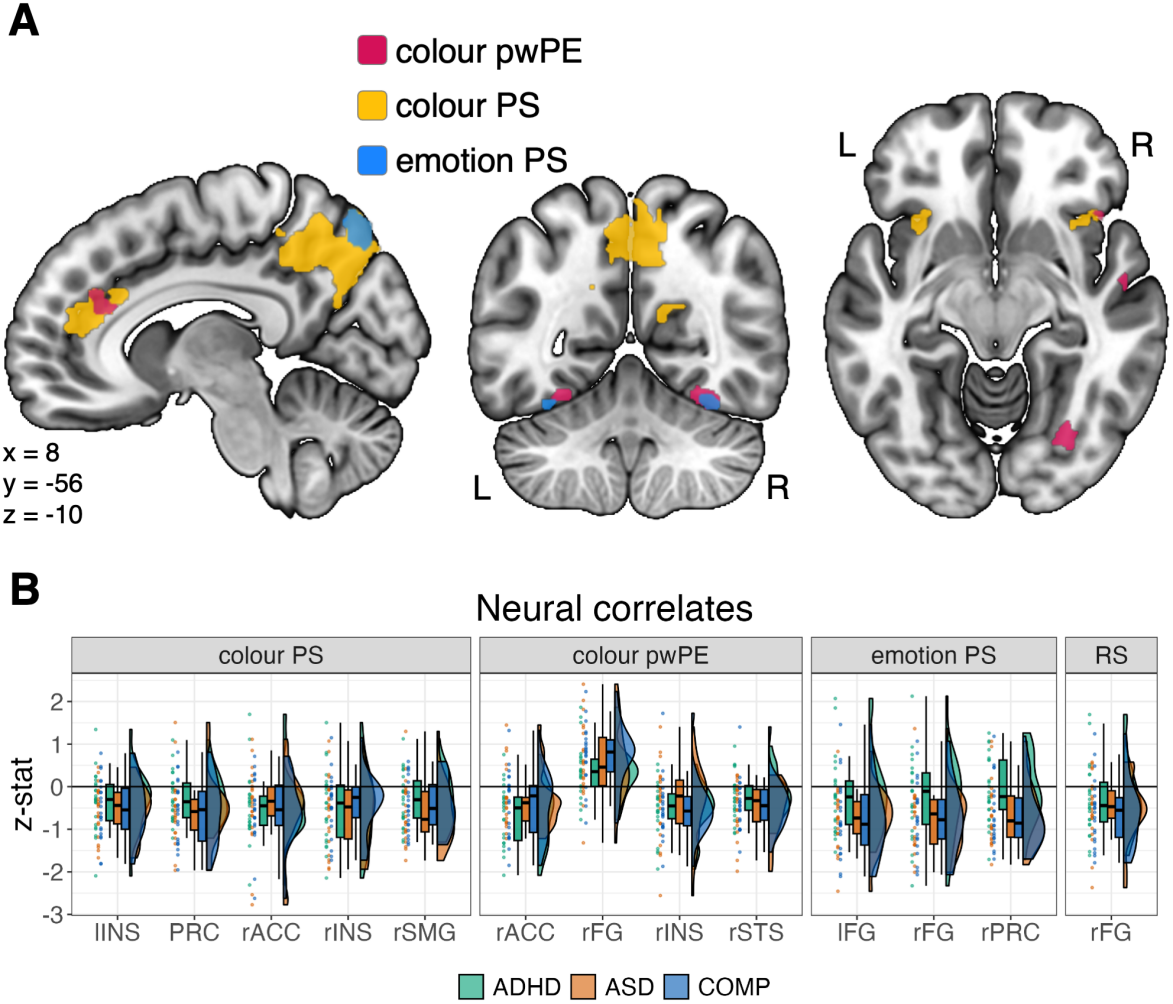
Results from the ROI analysis in the pooled sample showing neural correlates of precision-weighted prediction error (pwPE) and prediction strength (PS) as well as of repetition suppression (RS). Significance was assessed using threshold-free cluster enhancement and family-wise error correction with α = 0.05. (A) The location of the neural correlates of PS and pwPE, (B) participant-specific z-stats focusing on the largest clusters of each ROI separately for the three groups. *Note.* ACC = anterior cingulate cortex; ADHD = adults with attention-deficit/hyperactivity disorder; ASD = adults with autism spectrum disorder; COMP = comparison group of adults without psychiatric diagnoses; FG = fusiform gyrus; INS = insula; l = left; PRC = precuneus; r = right; SMG = supramarginal gyrus; STS = superior temporal sulcus.

In our hypothesis-guided ROI analysis of the COMP group, we found neural correlates of colour pwPE in the right fusiform gyrus (cluster size = 241, *f*-stat_max_ = 24.3, MNI_max_ = [30 -70 -10]; full list of local maxima provided in the Supplementary Materials S5.3) as well as neural correlates of emotion PS in the right precuneus (cluster size = 3, *f*-stat_max_ = 26.0, MNI_max_ = [6 -76 52]). Contrary to our hypothesis, we did not find any significant clusters associated with emotion pwPE in the COMP group in our ROI-guided analysis (p > = 0.497). We found a significant cluster in the fusiform gyrus associated with colour PS in the COMP group (two clusters, total cluster size = 18, *t*-stat_max_ = 28.8, MNI_max_ = [34 -38 -26]), diverging from results reported in Stefanics et al. (2019). Furthermore, the ROI analysis considering neural correlates in the pooled sample, combining all three groups, revealed several significant clusters of neural activation associated with colour PS and pwPE as well as emotion PS, but not emotion pwPE (see Table 2 and Fig. 2).

**Table 2.** Significant clusters in the ROI analysis exploring neural correlates of pwPE and PS in the pooled sample.

Region	Cluster size	H	*f*-value	x	y	z
*Colour pwPE*						
Fusiform gyrus	230	R	39.8	32	-60	-16
		R	35.9	28	-70	-12
		R	24.5	34	-74	-14
Superior temporal gyrus Insula	58	R	19.2	50	-40	22
	57	R	23.3	32	24	-4
		R	22.3	42	26	-4
		R	16.1	42	22	-10
Anterior cingulate cortex, supracallosal	47	R	22.0	8	34	24
Fusiform gyrus	28	L	32.1	-28	-56	-16
Superior temporal gyrus	15	R	17.9	52	-6	-14
Superior temporal gyrus	2	R	13.9	50	-18	-8
*Colour PS*						
Precuneus	2401	L	30.5	-8	-70	40
		R	28.0	12	-46	42
		R	27.7	8	-50	42
		R	26.8	4	-56	50
		R	25.2	10	-50	46
		R	24.1	2	-62	48
Supramarginal gyrus extending into the superior temporal gyrus	553	R	25.5	58	-44	36
		R	24.4	58	-46	44
		R	22.9	48	-40	10
		R	21.9	52	-44	38
		R	21.2	52	-40	44
		R	20.4	58	-50	18
Anterior cingulate cortex, supracallosal and pregenual	254	R	22.4	10	24	26
		R	20.9	8	40	16
		R	15.1	8	46	14
Insula	171	L	23.3	-28	22	4
		L	22.4	-32	20	-8
		L	21.2	-28	26	-4
		L	16.8	-36	16	-2
Insula	119	R	26.9	32	24	-4
		R	24.2	38	26	-4
		R	22.1	42	20	-8
		R	14.6	44	22	0
Precuneus	63	R	13.1	20	-54	20
		R	11.9	14	-54	18
Precuneus	3	R	12.5	4	-46	14
*Emotion pwPE*						
No significant clusters.						
*Emotion PS*						
Precuneus	124	R	23.9	8	-78	56
		R	23.6	6	-74	52
		R	18.2	16	-70	48
Fusiform gyrus	86	R	30.1	32	-52	-20
		R	21.5	28	-52	-14
		R	20.4	32	-62	-18
		R	17.9	30	-62	-14
Fusiform gyrus	62	L	24.7	-30	-64	-16
		L	22.8	-34	-60	-18
		L	20.4	-34	-54	-18
		L	17.9	-34	-54	-22
Precuneus	2	R	13.2	14	-74	48

*Note*. H = hemisphere; PS = prediction strength; pwPE = precision-weighted prediction error.

### 3.3 Repetition suppression

As predicted by our hypothesis, we found a significant decrease in neural activation in the right fusiform gyrus with increased repetition of faces with the same colour and emotion combination (three clusters, total cluster size = 94, *t*-stat_max_ = 4.62, MNI_max_ = [38 -56 -22]; full list of local maxima is provided in the Supplementary Materials S5.3), suggesting neural adaptation in the form of RS (see Fig. 2). However, we found no significant clusters in any of the expected areas associated with emotion processing, in contrast to our hypothesis. Furthermore, we found no significant clusters in the explorative whole-brain analysis, neither for neural adaptation to faces of the same colour and emotion combination (p > = 0.126) nor for neural adaptation to faces of the same colour (p > = 0.102) or faces with the same emotion (p > = 0.114).

### 3.4 Differences in neural correlates between groups

We observed no significant differences in the neural correlates of PS, pwPE, and RS between the three groups, neither in the hypothesis-guided nor in the explorative whole-brain analyses and regardless of whether age and gender were added as covariates. Therefore, we reject the hypotheses that there are differences in neural adaptation of emotions between autistic and comparison adults as well as differences between colour or emotion pwPE between adults with ASD or ADHD and adults in a comparison group.

Bayesian ANOVAs of the extracted activation also revealed evidence against differences between the groups, with anecdotal evidence against a difference for emotion PS in the right precuneus (log(*BF_10_corrected_*) = -0.087) and the left fusiform gyrus (log(*BF_10_corrected_*) = -1.004), moderate evidence against a difference for emotion PS in the right fusiform gyrus (log(*BF_10_corrected_*) = -1.761), colour pwPE in the right anterior cingulate cortex (log(*BF_10_corrected_*) = -2.209), and colour PS in the precuneus (log(*BF_10_corrected_*) = -2.232), as well as strong evidence against all other differences (see the Supplementary Materials S5.4 for all Bayes Factors). Furthermore, a Bayesian ANOVA focusing on extracted activation from the right fusiform gyrus with the predictors group and feature (emotion or colour) revealed the model only including feature to best describe the data (log(*BF_10_corrected_*) = 7.311; see Supplementary Materials S5.5). There was moderate evidence in favour of this model compared with the second-best model which additionally included the predictor group (log(*BF_corrected_*) = 1.741).

### 3.5 Behavioural analyses

Our Bayesian linear mixed model with the hit reaction times as the outcome and group (ASD, ADHD, or COMP) as a predictor showed no credible differences, neither between the COMP and ADHD groups (CI of COMP—ADHD: -68.31 to 16.15 ms, posterior probability = 89.22%) nor the ASD and the COMP groups (CI of COMP—ASD: -49.3 to 33.53 ms, posterior probability = 66.11%). Similarly, our Bayesian linear mixed model with the negative discrimination rate (perfect discrimination rate—actual discrimination rate) as the outcome and group as a predictor likewise showed no credible differences between the groups (CI of COMP—ADHD: -9.72 to 7.92, posterior probability = 60.45%; CI of COMP—ASD: -2.97 to 11.87, posterior probability = 86.88%). Visualisations are provided in the Supplementary Materials S3.

Overall, centre dwell times were high with 93% for ADHD, 93% for ASD, and 91% for COMP. There was anecdotal evidence against differences due to group (log(*BF_10_*) = -0.040), suggesting that all three groups similarly focused on the centre, as instructed (see heatmaps in the Supplementary Materials S3.3.3).

## 4 Discussion

Our brain constantly interprets the sensory input it receives and uses it to make predictions about the world. In a visual mismatch paradigm, we induced predictions about two features, emotions and colours, of unattended, task-irrelevant face stimuli in adults with ASD or ADHD as well as a non-clinical comparison group. Repeated presentation of the same colour and emotion combination led to repetition suppression in the right fusiform gyrus. Furthermore, neural correlates of colour pwPE and PS were found in regions associated with face and emotion processing. While we did not find neural correlates of emotion pwPE, we found emotion PS to be associated with activation in the right precuneus and bilateral fusiform gyrus. Neural correlates of PS and pwPE induced by colour and emotion of faces were comparable between adults with ADHD or ASD and comparison participants.

We expected decreased neural adaptation to emotions as well as differences in neural correlates of pwPE of emotion and colour in autistic adults compared with adults without psychiatric diagnoses. These hypotheses were not supported by the observed data. These findings differ from those of a previous study showing decreased neural correlates of pwPE in autism (Sapey-Triomphe et al., 2023). There are significant task differences in terms of explicit versus implicit testing between the current study and the study conducted by Sapey-Triomphe and colleagues that could account for the discrepancy: the latter used an associative learning task where participants were asked to predict the rotation of two centrally presented dots based on a preceding tone. The probabilistic association between the tone cue and rotation direction changed over the course of the task. The authors found differences between autistic and non-autistic adults in their neural correlates of pwPE in the anterior cingulate cortex and the putamen. However, in their task, the stimuli eliciting the pwPE were both task relevant and attended, while the stimuli eliciting pwPE in the current task were task irrelevant and unattended in the periphery. The discrepancy in findings could indicate that explicit attention to and relevance of stimulus features might lead to pwPE modifications in autism, whereas implicit error processing might be spared. Future research could manipulate explicit attention with regard to hierarchical prediction errors to assess whether modifications associated with autism are modulated by explicit attention.

Previous studies revealed processing differences associated with autism to be features specific; for example, differences in RS were found for faces but not objects (Ewbank et al., 2017). Thus, we explored whether decreased neural correlates of pwPE might be associated with social information and, therefore, be domain specific in autism. While we did not observe any differences between the autistic and the comparison group in the current study, analyses of the pooled sample revealed only neural correlates for colour but not emotion pwPE, possibly suggesting that emotion presented in the periphery might not be salient enough to elicit a pwPE. Based on our data, we cannot conclude whether more salient presentation of the feature emotion might have led to differentiated neural correlates of emotion pwPE between autistic and comparison adults. Nonetheless, we found neural correlates of pwPE for the feature colour, all else being equivalent, in our pooled sample, but no modification in autistic adults. Therefore, our data suggest unaltered neural correlates of colour pwPE and possibly emotion pwPE in autistic adults viewing unattended faces. However, since no neural correlates for emotion pwPE were found in our sample, we will refrain from discussing group differences in emotion pwPE any further.

Comparable neural correlates of RS to colour and emotion combinations of faces in our autistic participants extend previous findings demonstrating reduced RS for faces in autism (D’Mello et al., 2023; Ewbank et al., 2017). It should be noted that our task design differed from these studies: instead of repeating facial identities, we repeated the colour and emotion of the faces. RS was elicited for colour and emotion combinations only, not for colour or emotion separately. The observed comparable activation patterns suggest that RS of shared features of faces, such as colour and emotion in this study, is unaltered in ASD, while RS for facial identities might be reduced (Ewbank et al., 2017). Furthermore, we used a task where the faces were presented in the periphery and irrelevant to the task, thus, encouraging our participants to not pay attention to the faces. While Ewbank et al. (2017) also employed a task unrelated to the stimuli of interest, they were still presented in the same visual field. Therefore, differences between RS in autistic and non-autistic adults might only emerge for stimuli within the attended visual field. While the exact role attention plays for RS is still unclear, there are some studies suggesting that attention but not awareness might be crucial for observing RS to faces in the fusiform gyrus (Henson, 2016). Autistic and non-autistic adults could potentially differ in how much they ignore a face appearing in their attended visual field if it is task irrelevant. This difference in the level of attention they pay to the face stimuli might be the driving factor behind the decreased RS to faces observed by Ewbank et al. (2017). In the current study, the placement in the periphery might have led to a similar degree of attention in both groups. Future research could assess this hypothesis by manipulating the distance between the stimuli leading to RS and the task-relevant stimuli.

Moreover, the hypothesised difference of colour and emotion pwPE between adults with ADHD and comparison adults was also not supported by the current data. This finding does not align with the literature showing reduced MMN in ADHD (Cheng et al., 2016; Dang et al., 2024; Hsieh et al., 2021; Kim et al., 2021), which has been interpreted as a marker of pwPE (Stefanics et al., 2014). However, neural correlates assessed using neuroimaging data differ fundamentally from neurophysiological data, as the temporal resolution is much lower. Decreased pwPE in ADHD might, therefore, only apply to brief processes as captured by the MMN. Additionally, most studies investigating the MMN in adults with ADHD have focused on auditory MMN (Hsieh et al., 2021) or non-social visual stimuli (Dang et al., 2024). Furthermore, a significant number of the adults with ADHD tested in the current study were medicated with 17% taking amphetamines and almost 35% taking methylphenidate. These stimulants affect striato-cortical regions by modulating norepinephrine and dopamine (Faraone, 2018). Studies using acute administration of stimulants demonstrated that neural activity in adults with ADHD can be modulated towards similar levels as those of adults without ADHD in various tasks (Parlatini et al., 2024). Thus, the large proportion of adults taking stimulants in our sample might have decreased any potential differences between our ADHD and our comparison group. However, our sample showed differences on behavioural tasks outside of the scanner, as detailed in the next paragraph. Therefore, the absence of differences between adults with ADHD and comparison adults with regard to colour and possibly emotion pwPE indicates unaltered predictive processes in ADHD for low-level visual and possibly complex social information.

Task performance of our clinical groups and the comparison group did not differ credibly nor did their attentional focus as assessed through dwell times. However, the participants in the clinical and comparison groups also completed behavioural tasks outside of the scanner where we found credible differences between the groups, including increased discrimination thresholds for recognising emotions based on facial expressions (Plank et al., 2024). Most importantly, participants completed an associative learning task based on which we extracted participant-specific belief state trajectories using a three-level HGF (Plank, Pior et al., 2025). Neither parameters of the perceptual nor of the response model differed credibly between adults with ASD or ADHD and adults in the comparison group. Therefore, we conclude that our clinical samples were sufficiently different from our comparison group on the behavioural level in some processes, namely face attention bias and facial emotion recognition, but did not differ from the comparison groups in terms of associative learning.

In line with our hypothesis, we found neural correlates of colour pwPE in the right but not the left fusiform gyrus in our comparison group when focusing on the bilateral fusiform gyrus. When assessing neural correlates of colour pwPE in the pooled sample in areas associated with face and emotion processing, we found additional clusters in the right superior temporal gyrus, the right insula, the right anterior cingulate cortex, and the left fusiform gyrus. Visual inspection of participant-specific *z*-stats indicates an increase in activation in the right fusiform gyrus and a decrease in the other areas. This finding partially replicates Stefanics et al. (2019), who detected neural correlates of colour pwPE in adults without psychiatric diagnoses in the bilateral fusiform gyrus. Both in the original paper by Stefanics et al. (2019) and in the current data, visual inspection of the contrast estimates indicates an increase of activation in association with colour pwPE. However, Stefanics et al. (2019) did not report neural correlates of colour PS, which contrasts the current finding of widespread neural correlates of colour PS in the pooled sample. These neural correlates of colour PS were found in the bilateral precuneus, right superior temporal gyrus extending into the supramarginal gyrus, right anterior cingulate cortex, and bilateral insula; thus, we detected neural correlates of colour PS in areas associated with emotion processing. The design of the task might have facilitated this association by using coloured emotional faces where the colour was possibly more salient. However, the stimuli were the same as in Stefanics et al. (2019), thus, not explaining the discrepancy of findings. Furthermore, colours are commonly associated with emotions, so much so that the popular depiction of emotions in Pixar’s movie *Inside Out* heavily draws on coloured representation. This has also been demonstrated using empirical studies. Specifically, Thorstenson et al. (2018) found that redness was more strongly associated with happiness while greenness was more strongly associated with fear. Therefore, colour in the current task design might not have been perceived as emotionally neutral.

We found evidence regarding neural correlates of emotion PS in areas associated with emotion processing in our comparison group. Specifically, we found decreased activation in the right precuneus with increasing emotion PS. In the pooled sample, we additionally found neural correlates of emotion PS in the bilateral fusiform gyrus with visual inspection indicating a decrease in activation associated with emotion PS. The latter finding is comparable with the findings of Stefanics et al. (2019); however, they report an additional cluster in the right superior temporal gyrus but no associated activation in the precuneus. Overall, the similarity in results suggests converging evidence of a prominent role of the fusiform gyrus in tracking emotion-specific predictions associated with faces.

Based on the findings of Stefanics et al. (2019), we hypothesised neural correlates of emotion pwPE in areas associated with emotion processing, including the right supramarginal gyrus and the bilateral precuneus. However, this hypothesis was not supported in our data. In fact, we did not find any neural correlates of emotion pwPE, possibly suggesting that the emotional expressions of the faces were not salient enough to elicit pwPE in the case of mismatch, although predictions emerged as indicated by the neural correlates of emotion PS in the precuneus and the fusiform gyrus. We can only speculate why our findings differ from those of the original paper. The original sample was more homogeneous in terms of age than our comparison group. Additionally, they encountered twice as many tetrads as participants in the current study as we shortened the paradigm to adjust it to the needs of our clinical groups. Future research should assess neural correlates of emotion pwPE in large sample of adults and consider its relation to emotion PS. We would expect neural encoding of pwPE to complement neural encoding of PS: with increasing PS, the strength of the pwPE when input contrasts the prediction is increasing as well. However, we only found evidence of neural encoding of PS but not pwPE, while Stefanics et al. (2019) observed the opposite pattern.

While our comparison sample captured a wide range of ages and education levels, our clinical samples were more homogeneous and our conclusions are thus subject to certain constraints on generality (Simons et al., 2017). First, we excluded all adults with a double diagnosis of ASD and ADHD in this study. The possibility of a double diagnosis has been added to the newest iteration of the International Classification of Diseases (Taurines et al., 2012; World Health Organization, 2019), and estimates for ADHD in autistic adults have been reported as high as 28% (Lai et al., 2019). Since we only included adults with either a diagnosis of ASD or a diagnosis of ADHD, we cannot generalise to those with co-occurring ASD and ADHD, which are a significant portion of autistic adults. However, due to the recent change in diagnostic criteria, we cannot rule out that some of our participants may fulfil the criteria for the comorbid diagnoses but were never assessed for it. While many of the participants with ADHD scored above the cut-off for the RADS-R, it is important to note that neither the RADS-R nor the ASRS-v1.1 is specific enough to indicate a diagnosis on their own (Kessler et al., 2005; Rausch et al., 2024). Furthermore, only verbal adults without intellectual disability were included in the current study, who might not be representative for the whole spectrum. Concerning the sampled ADHD group, we included both medicated and unmedicated adults. While this pattern is representative of the ADHD population, it might have affected performance. Furthermore, all participants in this study were between 16 and 45 years old. Thus, we cannot assess whether our results generalise to older adults. Last, while we did achieve our preregistered sample size of analysing 22 participants per group, the comparison sample of adults without psychiatric diagnoses was smaller than in the original article by Stefanics et al. (2019) where participants also encountered eight instead of four runs; thus, our study might have been underpowered to detect certain effects.

In conclusion, we found that neural correlates of pwPE for low-level visual features remain intact in ASD and ADHD. Unattended, task-irrelevant faces did not yield neural differences across adults with ASD, adults with ADHD, and adults without any psychiatric diagnoses, regardless of whether the predictions related to low-level visual features or complex social information. Thus, these findings imply that while explicit Bayesian processes may falter in neurodevelopmental disorders, implicit task-irrelevant processing remains intact.

## Supplementary Material

Supplementary Material

## Data Availability

We share preprocessed and anonymised behavioural data, anonymised contrast files from the subject-level of FSL, and all code used in this project on GitHub: https://github.com/IreneSophia/EMBA_VMM.
