# Supplementary material for: Are prediction error modifications domain specific in autism but domain general in ADHD?
Source: Imaging Neurosci (Camb). 2025 Oct 17;3:IMAG.a.942. doi: 10.1162/IMAG.a.942 (PMC12534711; doi:10.1162/IMAG.a.942)
Supplement: Supplementary Material [file IMAG.a.942_supp.pdf]

SUPPLEMENTARY MATERIALS

Are prediction error attenuations domain-specific in autism but domain-general in ADHD?

I S Plank et al.

2025-07-28

Contents

1 Packages and load data **1**

2 Participants **2**

3 Behavioural and eye-tracking results **3**
3.1 Reaction times 4
3.1.1 Model and inferences 4
3.1.2 Plots 6
3.2 Discrimination rate 7
3.2.1 Model and inferences 7
3.2.2 Plots 10
3.3 Analysis of fixation proportions to centre AOI 11
3.3.1 Bayesian ANOVAs 11
3.3.2 Plots 12
3.3.3 Heatmaps 13

4 fMRPrep **17**
4.1 Preprocessing of B0 inhomogeneity mappings 17
4.2 Anatomical data preprocessing 17
4.3 Functional data preprocessing 17
4.3.1 Copyright Waiver 18
4.3.2 References 18

5 FSL analysis **20**
5.1 Regions of interest 20
5.2 Combine FSL output 20
5.3 Hypothesis-guided ROI analysis 21
5.4 Plotting 23
5.5 Focusing on fusiform gyrus 26

1 Packages and load data

```
## [1] "R version 4.5.1 (2025-06-13)"

## [1] "knitr version 1.50"
## [1] "ggplot2 version 3.5.2"
## [1] "tidyverse version 2.0.0"
## [1] "ggpubr version 0.6.1"
## [1] "brms version 2.22.0"
```

```
## [1] "ggrain version 0.0.4"
## [1] "BayesFactor version 0.9.12.4.7"
## [1] "effectsize version 1.0.1"
## [1] "vtable version 1.4.8"
## [1] "rstatix version 0.7.2"
## [1] "bayestestR version 0.16.1"
```

```
## `summarise()` has grouped output by 'subID'. You can override using the
## `.groups` argument.
```

2 Participants

```
# print the group of included participants
kable(df.disc %>% select(subID, diagnosis) %>% distinct() %>%
        group_by(diagnosis) %>% count())
```

diagnosis	n
ADHD	23
ASD	22
COMP	22

```
# print the group of included participants with eye tracking
kable(df.fix.agg %>% select(subID, diagnosis) %>% distinct() %>%
        group_by(diagnosis) %>% count())
```

diagnosis	n
ADHD	16
ASD	11
CTR	15

```
# print the outcome of the contingency table concerning gender
ct.full@bayesFactor
```

```
##                           bf error                     time         code
## Non-indep. (a=1) -2.932155     0 Mon Feb 17 10:45:09 2025 2d6a747c15f0d
```

```
ct.mf@bayesFactor
```

```
##                           bf error                     time         code
## Non-indep. (a=1) -1.088224     0 Mon Feb 17 10:45:09 2025 2d6a77dfa2c24
```

```
# print the outcome of the contingency table concerning education
ct.edu@bayesFactor
```

```
##                           bf error                     time         code
## Non-indep. (a=1) -1.834707     0 Mon Feb 17 10:45:09 2025 2d6a731fddab1
```

```
# print the associated table
kable(as.data.frame(tb.edu) %>%
        pivot_wider(names_from = diagnosis, values_from = Freq) %>%
        mutate(
          description = case_match(edu,
                                   "1" ~ "No educational or vocational training",
```

```
                                      "2" ~ "Middle school education",
                                      "3" ~ "Trade school",
                                      "4" ~ "High school education",
                                      "5" ~ "University degree (Bachelor, Master, PhD)")
           )
       )
```

edu	ADHD	ASD	COMP	description
1	2	0	0	No educational or vocational training
2	1	2	1	Middle school education
3	5	5	2	Trade school
4	12	8	7	High school education
5	3	7	12	University degree (Bachelor, Master, PhD)

```
# print the outcome of the screening with ASRS and RADS
kable(tb.screen)
```

	screenADHD	screenASD	screenBOTH	screenNone
ADHD	5	1	12	5
ASD	1	16	4	1
COMP	1	3	0	18

```
# print medication for the paper
kable(df.meds)
```

diagnosis	adhd.meds	psych.meds_binary	n
ADHD	atomoxetine	TRUE	1
ADHD	lisdexamfetamine	TRUE	4
ADHD	methylphenidate	TRUE	8
ADHD	NA	FALSE	8
ADHD	NA	TRUE	2
ASD	NA	FALSE	11
ASD	NA	TRUE	11
COMP	NA	FALSE	22

3 Behavioural and eye-tracking results

```
# load the models
m.rtc = readRDS(file.path(model_dir, "m_rtc_agg.rds"))
m.disc = readRDS(file.path(model_dir, "m_disc.rds"))

# set and print the contrasts
contrasts(df.rtc.agg$diagnosis)  = contr.sum(3)
contrasts(df.rtc.agg$diagnosis)
```

```
##      [,1] [,2]
## ADHD    1    0
## ASD     0    1
## COMP   -1   -1
```

```
contrasts(df.disc$diagnosis) = contr.sum(3)
contrasts(df.disc$diagnosis)
```

```
##      [,1] [,2]
## ADHD    1    0
## ASD     0    1
## COMP   -1   -1
```

```
contrasts(df.fix.agg$diagnosis) = contr.sum(3)
contrasts(df.fix.agg$diagnosis)
```

```
##      [,1] [,2]
## ADHD    1    0
## ASD     0    1
## CTR    -1   -1
```

3.1 Reaction times

3.1.1 Model and inferences

```
# print a summary
summary(m.rtc)
```

```
## Loading required namespace: rstan

##  Family: shifted_lognormal
##   Links: mu = identity; sigma = identity; ndt = identity
## Formula: rtc ~ diagnosis
##    Data: df.rtc.agg (Number of observations: 67)
##   Draws: 4 chains, each with iter = 4500; warmup = 1500; thin = 1;
##          total post-warmup draws = 12000
##
## Regression Coefficients:
##            Estimate Est.Error l-95% CI u-95% CI Rhat Bulk_ESS Tail_ESS
## Intercept      5.60      0.15     5.32     5.91 1.00     3304     3077
## diagnosis1     0.05      0.04    -0.03     0.14 1.00     6186     6527
## diagnosis2    -0.01      0.04    -0.09     0.07 1.00     5902     5970
##
## Further Distributional Parameters:
##       Estimate Est.Error l-95% CI u-95% CI Rhat Bulk_ESS Tail_ESS
## sigma     0.28      0.05     0.19     0.39 1.00     3454     3957
## ndt     145.29     40.12    52.77   209.99 1.00     3231     3147
##
## Draws were sampled using sample(hmc). For each parameter, Bulk_ESS
## and Tail_ESS are effective sample size measures, and Rhat is the potential
## scale reduction factor on split chains (at convergence, Rhat = 1).
```

```
# get the estimates and compute groups
df.m.rtc = as_draws_df(m.rtc) %>%
  select(starts_with("b_")) %>%
  mutate(
    b_COMP    = - b_diagnosis1 - b_diagnosis2,
    b_ASD       = b_diagnosis2,
    b_ADHD      = b_diagnosis1
    )
```

```
## Warning: Dropping 'draws_df' class as required metadata was removed.
```

```
# plot the posterior distributions
df.m.rtc %>%
  select(b_ASD, b_ADHD, b_COMP) %>%
  pivot_longer(cols = c(b_ASD, b_ADHD, b_COMP), names_to = "coef", values_to = "estimate") %>%
  group_by(coef) %>%
  mutate(
    cred = case_when(
      (mean(estimate) < 0 & quantile(estimate, probs = 0.975) < 0) |
        (mean(estimate) > 0 & quantile(estimate, probs = 0.025) > 0) ~ "credible",
      T ~ "not credible"
    )
  ) %>% ungroup() %>%
  ggplot(aes(x = estimate, y = coef, fill = cred)) +
  geom_vline(xintercept = 0, linetype = 'dashed') +
  ggdist::stat_halfeye(alpha = 0.7) + ylab(NULL) + theme_bw() +
  scale_fill_manual(values = c(credible = c_dark, c_light)) +
  theme(legend.position = "none")
```

```
## Warning: No shared levels found between `names(values)` of the manual scale and the
## data's fill values.
```

```
## Warning: No shared levels found between `names(values)` of the manual scale and the
## data's fill values.
```

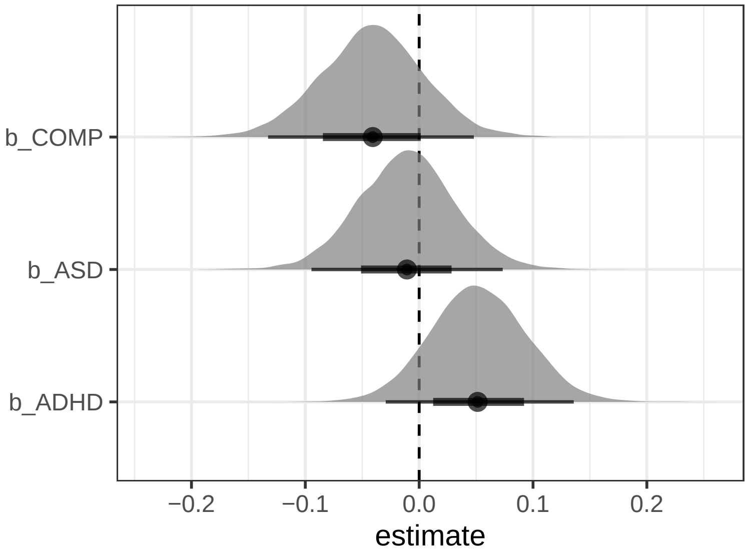


```
# ADHD slower than COMP
e1 = hypothesis(m.rtc, "0 < 2*diagnosis1 + diagnosis2", alpha = 0.025)
e1
```

```
## Hypothesis Tests for class b:
##                  Hypothesis Estimate Est.Error CI.Lower CI.Upper Evid.Ratio
## 1 (0)-(2*diagnosis1... < 0    -0.09      0.08    -0.25     0.06       8.28
##   Post.Prob Star
## 1      0.89
## ---
## 'CI': 95%-CI for one-sided and 97.5%-CI for two-sided hypotheses.
## '*': For one-sided hypotheses, the posterior probability exceeds 97.5%;
## for two-sided hypotheses, the value tested against lies outside the 97.5%-CI.
```

```
## Posterior probabilities of point hypotheses assume equal prior probabilities.
```

```
# ASD slower than COMP
e2 = hypothesis(m.rtc, "0 < 2*diagnosis2 + diagnosis1", alpha = 0.025)
e2
```

```
## Hypothesis Tests for class b:
##                 Hypothesis Estimate Est.Error CI.Lower CI.Upper Evid.Ratio
## 1 (0)-(2*diagnosis2... < 0    -0.03      0.08    -0.18     0.12       1.95
##   Post.Prob Star
## 1      0.66
## ---
## 'CI': 95%-CI for one-sided and 97.5%-CI for two-sided hypotheses.
## '*': For one-sided hypotheses, the posterior probability exceeds 97.5%;
## for two-sided hypotheses, the value tested against lies outside the 97.5%-CI.
## Posterior probabilities of point hypotheses assume equal prior probabilities.
```

```
# extract predicted differences in ms instead of log data
df.new = df.rtc.agg %>%
  select(diagnosis) %>%
  distinct()
df.ms = as.data.frame(
  fitted(m.rtc, summary = F,
             newdata = df.new %>% select(diagnosis),
             re_formula = NA))
colnames(df.ms) = df.new$diagnosis

# calculate our difference columns
df.ms = df.ms %>%
  mutate(
    COMP_ADHD  = COMP - ADHD,
    COMP_ASD   = COMP - ASD
  )
```

Our Bayesian linear mixed model with the hit reaction times as the outcome and diagnostic status as a predictor showed no credible differences: COMP participants reacted similarly to the ADHD group (CI of COMP - ADHD: -68.31 to 16.15ms, posterior probability = 89.22%) and the ASD group (CI of COMP - ASD: -49.3 to 33.53ms, posterior probability = 66.11%).

3.1.2 Plots

```
# overall median reaction times
df.rtc.agg %>%
  ggplot(aes(diagnosis, rtc, fill = diagnosis, colour = diagnosis)) + #
  geom_rain(rain.side = 'r',
boxplot.args = list(color = "black", outlier.shape = NA, show_guide = FALSE, alpha = 0.5),
violin.args = list(color = "black", outlier.shape = NA, alpha = 0.5),
boxplot.args.pos = list(
  position = ggpp::position_dodgenudge(x = 0, width = 0.3), width = 0.3
),
point.args = list(show_guide = FALSE, alpha = .5),
violin.args.pos = list(
  width = 0.6, position = position_nudge(x = 0.16)),
point.args.pos = list(position = ggpp::position_dodgenudge(x = -0.25, width = 0.1))) +
  ylim(0, 800) +
  scale_fill_manual(values = custom.col) +
```

```r
  scale_color_manual(values = custom.col) +
  labs(title = "Median reaction times per subject",
       x = "",
       y = "reaction time (ms)") +
  theme_bw() +
  theme(legend.position = "none",
        plot.title = element_text(hjust = 0.5),
        legend.direction = "horizontal",
        text = element_text(size = 15))
```

```
## Warning: The `show_guide` argument of `layer()` is deprecated as of ggplot2 2.0.0.
## i Please use the `show.legend` argument instead.
## i The deprecated feature was likely used in the ggrain package.
##   Please report the issue at <https://github.com/njudd/ggrain/issues>.
## This warning is displayed once every 8 hours.
## Call `lifecycle::last_lifecycle_warnings()` to see where this warning was
## generated.

## Warning in (function (mapping = NULL, data = NULL, stat = "half_ydensity", :
## Ignoring unknown parameters: `outlier.shape`
```

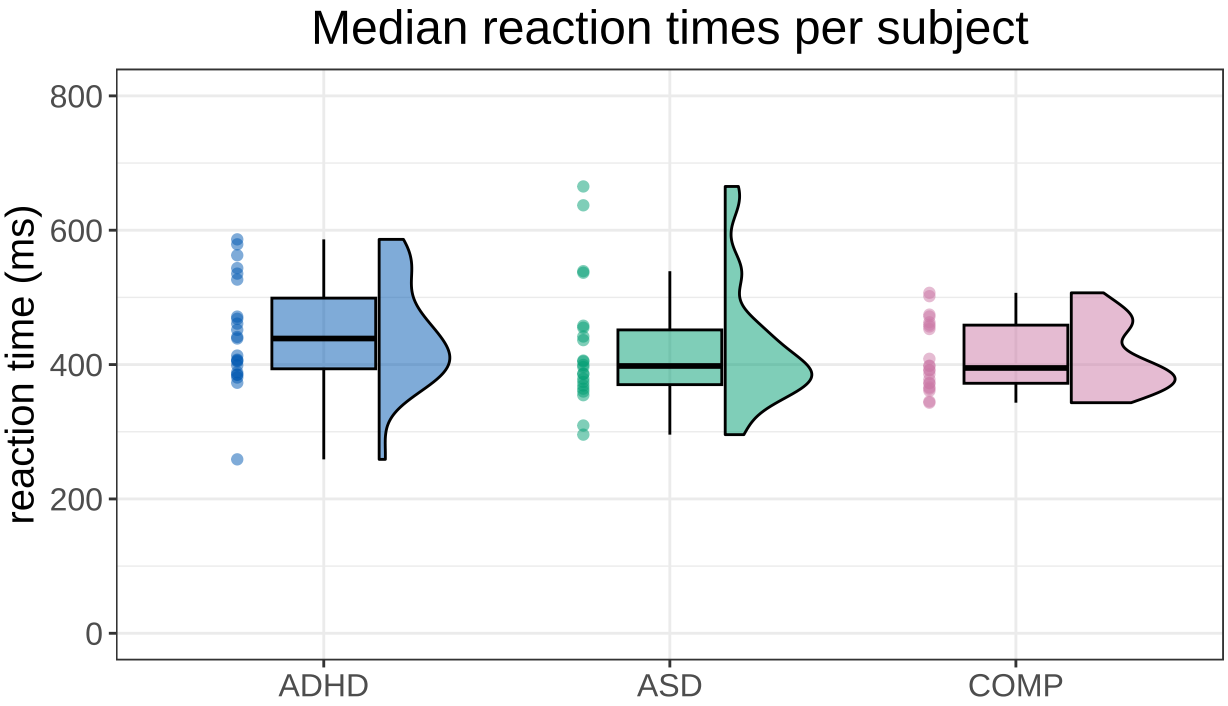

3.2 Discrimination rate

3.2.1 Model and inferences

```r
# print a summary
summary(m.disc)
```

```
##  Family: poisson
##   Links: mu = log
## Formula: negdisc ~ diagnosis + (1 | subID)
##    Data: df.disc (Number of observations: 67)
```

```
##   Draws: 4 chains, each with iter = 9000; warmup = 3000; thin = 1;
##          total post-warmup draws = 24000
##
## Multilevel Hyperparameters:
## ~subID (Number of levels: 67)
##               Estimate Est.Error l-95% CI u-95% CI Rhat Bulk_ESS Tail_ESS
## sd(Intercept)     1.04      0.11     0.85     1.28 1.00     4753     8889
##
## Regression Coefficients:
##            Estimate Est.Error l-95% CI u-95% CI Rhat Bulk_ESS Tail_ESS
## Intercept      2.43      0.14     2.16     2.69 1.00     4101     7578
## diagnosis1     0.18      0.19    -0.19     0.54 1.00     3261     6317
## diagnosis2    -0.27      0.19    -0.65     0.10 1.00     3529     6414
##
## Draws were sampled using sample(hmc). For each parameter, Bulk_ESS
## and Tail_ESS are effective sample size measures, and Rhat is the potential
## scale reduction factor on split chains (at convergence, Rhat = 1).
```

```
# get the estimates and compute groups
df.m.disc = as_draws_df(m.disc) %>%
  select(starts_with("b_")) %>%
  mutate(
    b_COMP    = - b_diagnosis1 - b_diagnosis2,
    b_ASD       = b_diagnosis2,
    b_ADHD      = b_diagnosis1
    )
```

```
## Warning: Dropping 'draws_df' class as required metadata was removed.
```

```
# plot the posterior distributions
df.m.disc %>%
  select(b_ASD, b_ADHD, b_COMP) %>%
  pivot_longer(cols = c(b_ASD, b_ADHD, b_COMP), names_to = "coef", values_to = "estimate") %>%
  group_by(coef) %>%
  mutate(
    cred = case_when(
      (mean(estimate) < 0 & quantile(estimate, probs = 0.975) < 0) |
        (mean(estimate) > 0 & quantile(estimate, probs = 0.025) > 0) ~ "credible",
      T ~ "not credible"
    )
  ) %>% ungroup() %>%
  ggplot(aes(x = estimate, y = coef, fill = cred)) +
  geom_vline(xintercept = 0, linetype = 'dashed') +
  ggdist::stat_halfeye(alpha = 0.7) + ylab(NULL) + theme_bw() +
  scale_fill_manual(values = c(credible = c_dark, c_light)) +
  theme(legend.position = "none")
```

```
## Warning: No shared levels found between `names(values)` of the manual scale and the
## data's fill values.
```

```
## Warning: No shared levels found between `names(values)` of the manual scale and the
## data's fill values.
```

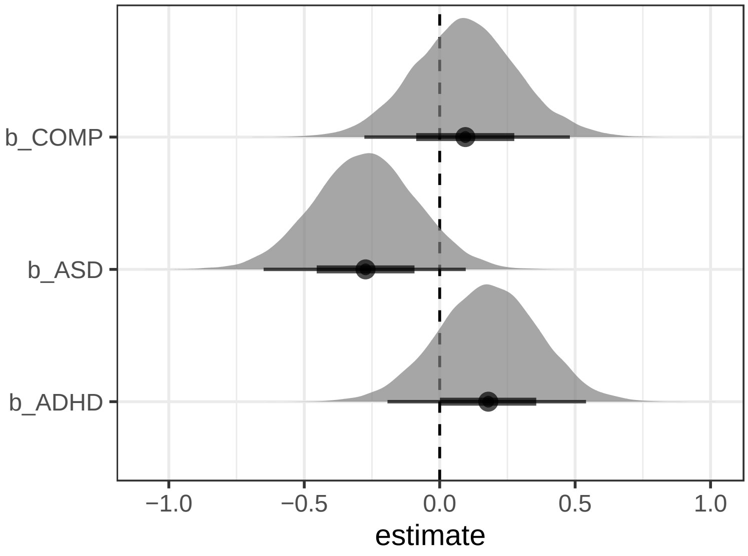


```r
# ADHD worse discrimination than COMP
e1 = hypothesis(m.disc, "0 < 2*diagnosis1 + diagnosis2", alpha = 0.025)
e1
```

```
## Hypothesis Tests for class b:
##                 Hypothesis Estimate Est.Error CI.Lower CI.Upper Evid.Ratio
## 1 (0)-(2*diagnosis1... < 0    -0.08      0.33    -0.72     0.58       1.53
##   Post.Prob Star
## 1       0.6
## ---
## 'CI': 95%-CI for one-sided and 97.5%-CI for two-sided hypotheses.
## '*': For one-sided hypotheses, the posterior probability exceeds 97.5%;
## for two-sided hypotheses, the value tested against lies outside the 97.5%-CI.
## Posterior probabilities of point hypotheses assume equal prior probabilities.
```

```r
# ASD worse discrimination than COMP
e2 = hypothesis(m.disc, "0 > 2*diagnosis2 + diagnosis1", alpha = 0.025)
e2
```

```
## Hypothesis Tests for class b:
##                 Hypothesis Estimate Est.Error CI.Lower CI.Upper Evid.Ratio
## 1 (0)-(2*diagnosis2... > 0     0.37      0.33    -0.28     1.03       6.62
##   Post.Prob Star
## 1      0.87
## ---
## 'CI': 95%-CI for one-sided and 97.5%-CI for two-sided hypotheses.
## '*': For one-sided hypotheses, the posterior probability exceeds 97.5%;
## for two-sided hypotheses, the value tested against lies outside the 97.5%-CI.
## Posterior probabilities of point hypotheses assume equal prior probabilities.
```

```r
# extract predicted differences in ms instead of log data
df.new = df.disc %>%
  select(diagnosis) %>%
  distinct()
df.ms = as.data.frame(
  fitted(m.disc, summary = F,
             newdata = df.new %>% select(diagnosis),
             re_formula = NA))
```

```r
colnames(df.ms) = df.new$diagnosis

# calculate our difference columns
df.ms = df.ms %>%
  mutate(
    COMP_ADHD  = COMP - ADHD,
    COMP_ASD   = COMP - ASD
  )
```

Our Bayesian linear mixed model with the negative discrimination rate (perfect discrimination rate - actual discrimination rate) as the outcome and diagnostic status as a predictor showed no credible differences: COMP participants reacted similarly to the ADHD group (CI of COMP - ADHD: -9.72 to 7.92ms, posterior probability = 60.45%) and the ASD group (CI of COMP - ASD: -2.97 to 11.87ms, posterior probability = 86.88%).

3.2.2 Plots

```r
# overall median reaction times
df.disc %>%
  ggplot(aes(diagnosis, disc, fill = diagnosis, colour = diagnosis)) + #
  geom_rain(rain.side = 'r',
boxplot.args = list(color = "black", outlier.shape = NA, show_guide = FALSE, alpha = 0.5),
violin.args = list(color = "black", outlier.shape = NA, alpha = 0.5),
boxplot.args.pos = list(
  position = ggpp::position_dodgenudge(x = 0, width = 0.3), width = 0.3
),
point.args = list(show_guide = FALSE, alpha = .5),
violin.args.pos = list(
  width = 0.6, position = position_nudge(x = 0.16)),
point.args.pos = list(position = ggpp::position_dodgenudge(x = -0.25, width = 0.1))) +
  ylim(0, 240) +
  scale_fill_manual(values = custom.col) +
  scale_color_manual(values = custom.col) +
  labs(title = "Discrimination rate per subject",
       x = "",
       y = "") +
  theme_bw() +
  theme(legend.position = "none",
        plot.title = element_text(hjust = 0.5),
        legend.direction = "horizontal",
        text = element_text(size = 15))
```

```
## Warning in (function (mapping = NULL, data = NULL, stat = "half_ydensity", :
## Ignoring unknown parameters: `outlier.shape`
```

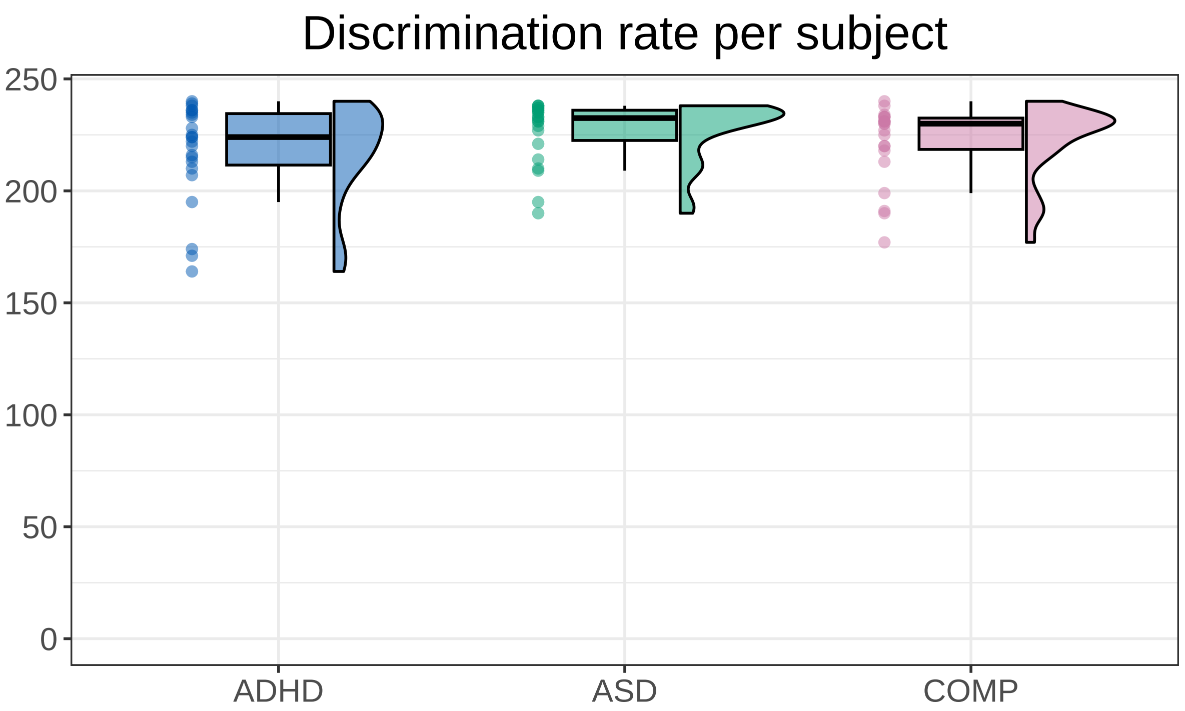

3.3 Analysis of fixation proportions to centre AOI

3.3.1 Bayesian ANOVAs

```
# check which outcomes of interest are normally distributed
df.fix.agg %>%
  group_by(diagnosis) %>%
  shapiro_test(fix.total, fix.prop, rfix.total, rfix.prop) %>%
  mutate(
    sig = if_else(p < 0.05, "*", "")
  ) %>% arrange(variable)
```

```
## # A tibble: 12 x 5
##    diagnosis variable   statistic           p sig
##    <fct>     <chr>          <dbl>       <dbl> <chr>
##  1 ADHD      fix.prop       0.580 0.0000107   "*"
##  2 ASD       fix.prop       0.443 0.000000317 "*"
##  3 CTR       fix.prop       0.538 0.00000725  "*"
##  4 ADHD      fix.total      0.905 0.0954      ""
##  5 ASD       fix.total      0.784 0.00586     "*"
##  6 CTR       fix.total      0.656 0.0000864   "*"
##  7 ADHD      rfix.prop      0.947 0.449       ""
##  8 ASD       rfix.prop      0.880 0.104       ""
##  9 CTR       rfix.prop      0.949 0.510       ""
## 10 ADHD      rfix.total     0.898 0.0738      ""
## 11 ASD       rfix.total     0.902 0.195       ""
## 12 CTR       rfix.total     0.948 0.491       ""
```

```
# ANOVA for the ranked proportional fixation durations
aov.fix = anovaBF(rfix.prop ~ diagnosis, data = df.fix.agg)
aov.fix@bayesFactor[["bf"]]
```

Table 6: Summary Statistics

diagnosis	ADHD			ASD			CTR		
Variable	N	Mean	SD	N	Mean	SD	N	Mean	SD
fix.centre	16	1040452	515022	11	1293957	467256	15	1308761	516135
fix.periphery	16	68752	74349	11	73310	172166	15	73488	132065
fix.total	16	1109204	513172	11	1367266	376425	15	1382249	495608
fix.prop	16	0.93	0.1	11	0.93	0.17	15	0.91	0.16

```
## [1] -0.04049587
```

```r
interpret_bf(aov.fix@bayesFactor[["bf"]], log = T)
```

```
## [1] "anecdotal evidence against"
## (Rules: jeffreys1961)
```

```r
# also explore if there are any differences in total fixation durations
aov.total = anovaBF(rfix.total ~ diagnosis, data = df.fix.agg)
aov.total@bayesFactor[["bf"]]
```

```
## [1] -0.575932
```

```r
interpret_bf(aov.total@bayesFactor[["bf"]], log = T)
```

```
## [1] "anecdotal evidence against"
## (Rules: jeffreys1961)
```

```r
# print some info on the raw values
st(df.fix.agg,
           vars = c('fix.centre', 'fix.periphery', 'fix.total', 'fix.prop'),
           group = 'diagnosis')
```

3.3.2 Plots

```r
# overall
df.fix.agg %>%
  ggplot(aes(diagnosis, fix.prop, fill = diagnosis, colour = diagnosis)) + #
  geom_rain(rain.side = 'r',
boxplot.args = list(color = "black", outlier.shape = NA, show_guide = FALSE, alpha = 0.5),
violin.args = list(color = "black", outlier.shape = NA, alpha = 0.5),
boxplot.args.pos = list(
  position = ggpp::position_dodgenudge(x = 0, width = 0.3), width = 0.3
),
point.args = list(show_guide = FALSE, alpha = .5),
violin.args.pos = list(
  width = 0.6, position = position_nudge(x = 0.16)),
point.args.pos = list(position = ggpp::position_dodgenudge(x = -0.25, width = 0.1))) +
  ylim(0.25, 1) +
  scale_fill_manual(values = custom.col) +
  scale_color_manual(values = custom.col) +
  labs(title = "Fixation proportions to centre of the screen",
       x = "",
       y = "% of fixation durations") +
  theme_bw() +
```

```
  theme(legend.position = "none",
        plot.title = element_text(hjust = 0.5),
        legend.direction = "horizontal",
        text = element_text(size = 15))
```

```
## Warning in (function (mapping = NULL, data = NULL, stat = "half_ydensity", :
## Ignoring unknown parameters: `outlier.shape`
```

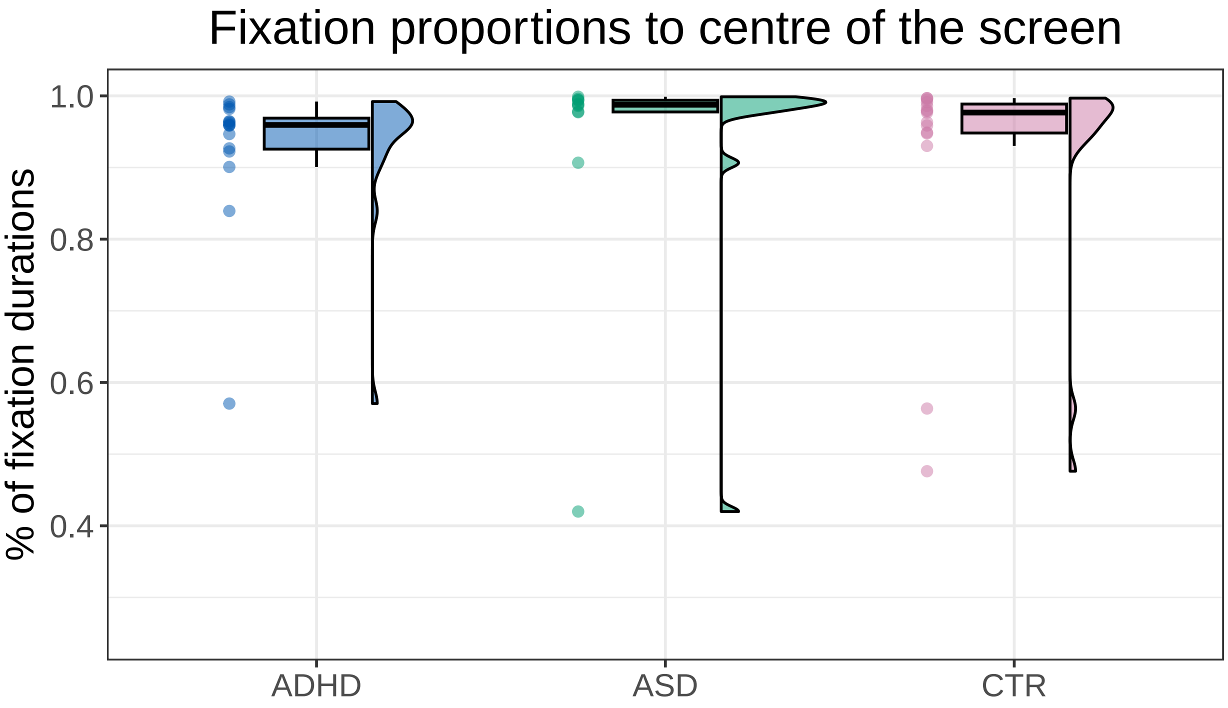

3.3.3 Heatmaps

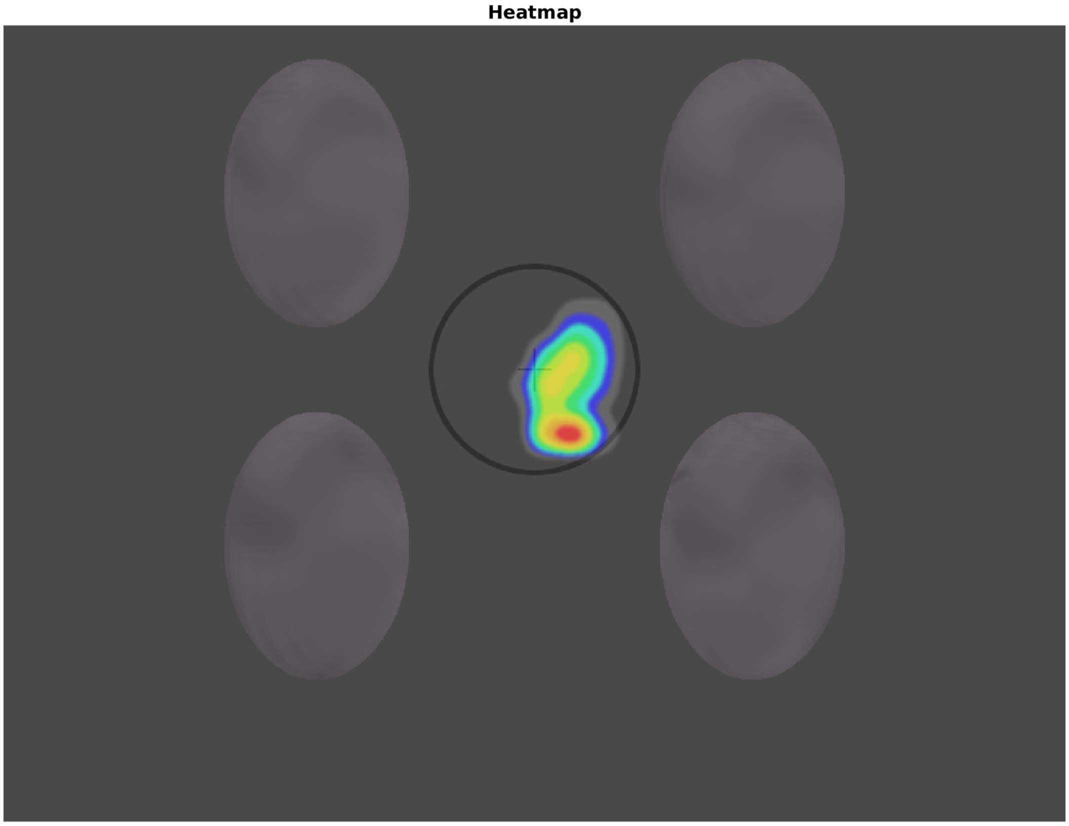

Figure 1: Heatmap of gaze pattern of the COMP group

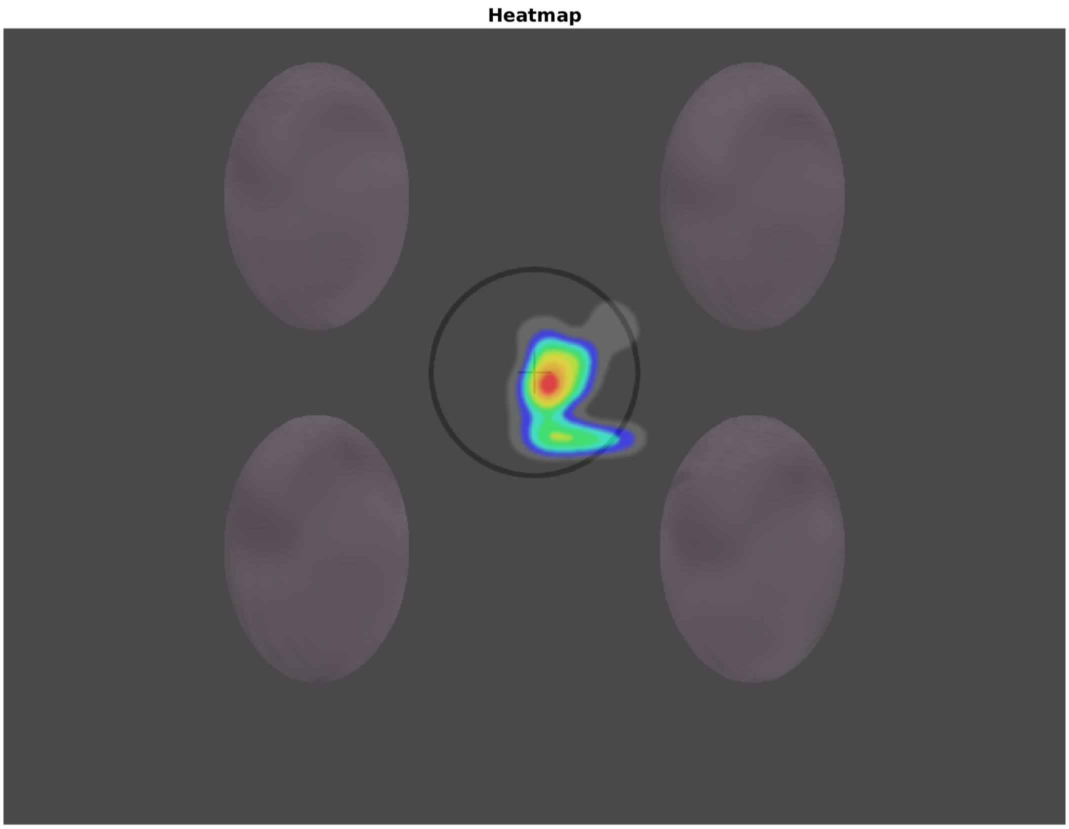

Figure 2: Heatmap of gaze pattern of the ADHD group

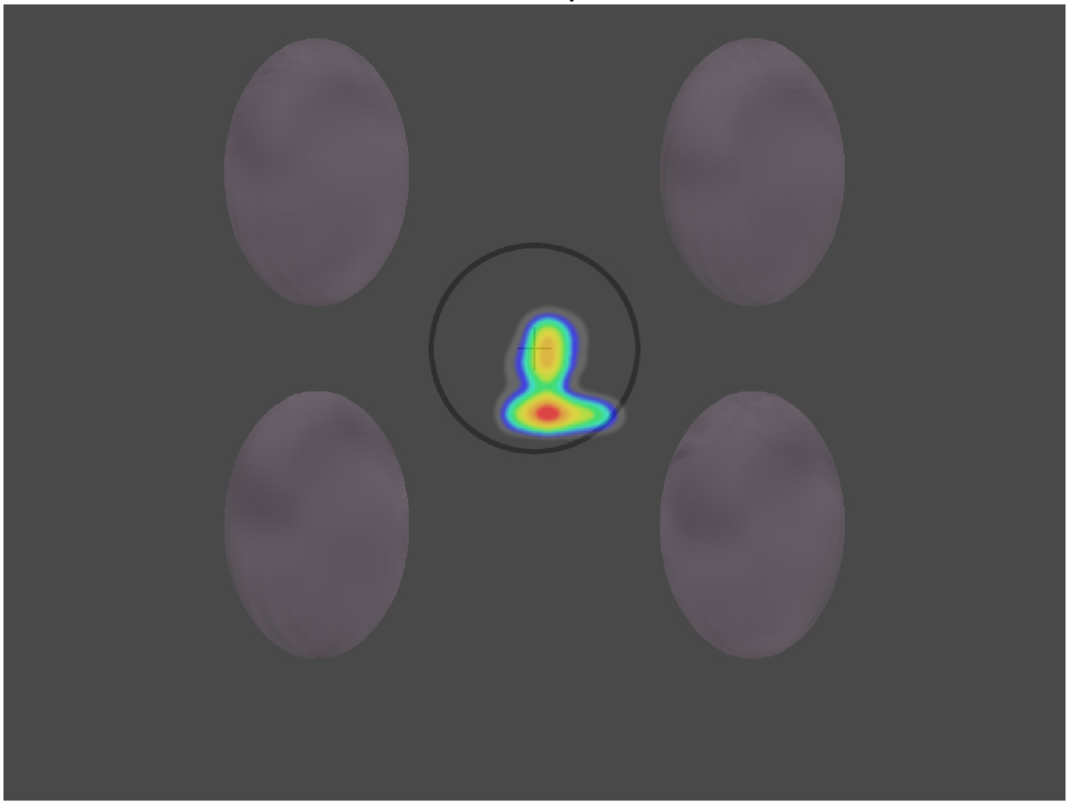

Figure 3: Heatmap of gaze pattern of the ASD group

4 fMRPrep

Results included in this manuscript come from preprocessing performed using *fMRIPrep* 23.0.2 (@fmriprep1; @fmriprep2; RRID:SCR_016216), which is based on *Nipype* 1.8.6 (@nipype1; @nipype2; RRID:SCR_002502).

4.1 Preprocessing of B0 inhomogeneity mappings

A total of 2 fieldmaps were found available within the input BIDS structure for this particular subject. A *B0*-nonuniformity map (or *fieldmap*) was estimated based on two (or more) echo-planar imaging (EPI) references with `topup` (@topup; FSL 6.0.5.1:57b01774).

4.2 Anatomical data preprocessing

A total of 2 T1-weighted (T1w) images were found within the input BIDS dataset. All of them were corrected for intensity non-uniformity (INU) with `N4BiasFieldCorrection` [@n4], distributed with ANTs 2.3.3 [@ants, RRID:SCR_004757]. The T1w-reference was then skull-stripped with a *Nipype* implementation of the `antsBrainExtraction.sh` workflow (from ANTs), using OASIS30ANTs as target template. Brain tissue segmentation of cerebrospinal fluid (CSF), white-matter (WM) and gray-matter (GM) was performed on the brain-extracted T1w using `fast` [FSL 6.0.5.1:57b01774, RRID:SCR_002823, @fsl_fast]. An anatomical T1w-reference map was computed after registration of 2 T1w images (after INU-correction) using `mri_robust_template` [FreeSurfer 7.3.2, @fs_template]. Brain surfaces were reconstructed using `recon-all` [FreeSurfer 7.3.2, RRID:SCR_001847, @fs_reconall], and the brain mask estimated previously was refined with a custom variation of the method to reconcile ANTs-derived and FreeSurfer-derived segmentations of the cortical gray-matter of Mindboggle [RRID:SCR_002438, @mindboggle]. Volume-based spatial normalization to two standard spaces (MNI152NLin6Asym, MNI152NLin2009cAsym) was performed through nonlinear registration with `antsRegistration` (ANTs 2.3.3), using brain-extracted versions of both T1w reference and the T1w template. The following templates were were selected for spatial normalization and accessed with *TemplateFlow* [23.0.0, @templateflow]: *FSL's MNI ICBM 152 non-linear 6th Generation Asymmetric Average Brain Stereotaxic Registration Model* [@mni152nlin6asym, RRID:SCR_002823; TemplateFlow ID: MNI152NLin6Asym], *ICBM 152 Nonlinear Asymmetrical template version 2009c* [@mni152nlin2009casym, RRID:SCR_008796; TemplateFlow ID: MNI152NLin2009cAsym].

4.3 Functional data preprocessing

For each of the 2 BOLD runs found per subject (across all tasks and sessions), the following preprocessing was performed. First, a reference volume and its skull-stripped version were generated using a custom methodology of *fMRIPrep*. Head-motion parameters with respect to the BOLD reference (transformation matrices, and six corresponding rotation and translation parameters) are estimated before any spatiotemporal filtering using `mcflirt` [FSL 6.0.5.1:57b01774, @mcflirt]. The estimated *fieldmap* was then aligned with rigid-registration to the target EPI (echo-planar imaging) reference run. The field coefficients were mapped on to the reference EPI using the transform. BOLD runs were slice-time corrected to 1.18s (0.5 of slice acquisition range 0s-2.37s) using `3dTshift` from AFNI [@afni, RRID:SCR_005927]. The BOLD reference was then co-registered to the T1w reference using `bbregister` (FreeSurfer) which implements boundary-based registration [@bbr]. Co-registration was configured with six degrees of freedom. Several confounding time-series were calculated based on the *preprocessed BOLD*: framewise displacement (FD), DVARS and three region-wise global signals. FD was computed using two formulations following Power (absolute sum of relative motions, @power_fd_dvars) and Jenkinson (relative root mean square displacement between affines, @mcflirt). FD and DVARS are calculated for each functional run, both using their implementations in *Nipype* [following the definitions by @power_fd_dvars]. The three global signals are extracted within the CSF, the WM, and the whole-brain masks. Additionally, a set of physiological regressors were extracted to allow for component-based noise correction [*CompCor*, @compcor]. Principal components are estimated after high-pass filtering the *preprocessed BOLD* time-series (using a discrete cosine filter with 128s cut-off) for the two *CompCor* variants: temporal (tCompCor) and anatomical (aCompCor). tCompCor components are then calculated from the top 2% variable voxels within the brain mask. For aCompCor, three probabilistic masks (CSF, WM and combined CSF+WM) are generated in anatomical space. The implementation differs

from that of Behzadi et al. in that instead of eroding the masks by 2 pixels on BOLD space, a mask of pixels that likely contain a volume fraction of GM is subtracted from the aCompCor masks. This mask is obtained by dilating a GM mask extracted from the FreeSurfer's *aseg* segmentation, and it ensures components are not extracted from voxels containing a minimal fraction of GM. Finally, these masks are resampled into BOLD space and binarized by thresholding at 0.99 (as in the original implementation). Components are also calculated separately within the WM and CSF masks. For each CompCor decomposition, the k components with the largest singular values are retained, such that the retained components' time series are sufficient to explain 50 percent of variance across the nuisance mask (CSF, WM, combined, or temporal). The remaining components are dropped from consideration. The head-motion estimates calculated in the correction step were also placed within the corresponding confounds file. The confound time series derived from head motion estimates and global signals were expanded with the inclusion of temporal derivatives and quadratic terms for each [@confounds_satterthwaite_2013]. Frames that exceeded a threshold of 0.5 mm FD or 1.5 standardized DVARS were annotated as motion outliers. Additional nuisance timeseries are calculated by means of principal components analysis of the signal found within a thin band (*crown*) of voxels around the edge of the brain, as proposed by [@patriat_improved_2017]. The BOLD time-series were resampled into standard space, generating a *preprocessed BOLD run in MNI152NLin6Asym space*. First, a reference volume and its skull-stripped version were generated using a custom methodology of *fMRIPrep*. Automatic removal of motion artifacts using independent component analysis [ICA-AROMA, @aroma] was performed on the *preprocessed BOLD on MNI space* time-series after removal of non-steady state volumes and spatial smoothing with an isotropic, Gaussian kernel of 6mm FWHM (full-width half-maximum). Corresponding "non-aggresively" denoised runs were produced after such smoothing. Additionally, the "aggressive" noise-regressors were collected and placed in the corresponding confounds file. All resamplings can be performed with *a single interpolation step* by composing all the pertinent transformations (i.e. head-motion transform matrices, susceptibility distortion correction when available, and co-registrations to anatomical and output spaces). Gridded (volumetric) resamplings were performed using `antsApplyTransforms` (ANTs), configured with Lanczos interpolation to minimize the smoothing effects of other kernels [@lanczos]. Non-gridded (surface) resamplings were performed using `mri_vol2surf` (FreeSurfer).

Many internal operations of *fMRIPrep* use *Nilearn* 0.9.1 [@nilearn, RRID:SCR_001362], mostly within the functional processing workflow. For more details of the pipeline, see the section corresponding to workflows in *fMRIPrep*'s documentation.

4.3.1 Copyright Waiver

The above boilerplate text was automatically generated by fMRIPrep with the express intention that users should copy and paste this text into their manuscripts *unchanged*. It is released under the CC0 license.

4.3.2 References

Abraham, Alexandre, Fabian Pedregosa, Michael Eickenberg, Philippe Gervais, Andreas Mueller, Jean Kossaifi, Alexandre Gramfort, Bertrand Thirion, and Gael Varoquaux. 2014. "Machine Learning for Neuroimaging with Scikit-Learn." Frontiers in Neuroinformatics 8. https://doi.org/10.3389/fninf.2014.00014.

Andersson, Jesper L. R., Stefan Skare, and John Ashburner. 2003. "How to Correct Susceptibility Distortions in Spin-Echo Echo-Planar Images: Application to Diffusion Tensor Imaging." NeuroImage 20 (2): 870–88. https://doi.org/10.1016/S1053-8119(03)00336-7.

Avants, B. B., C. L. Epstein, M. Grossman, and J. C. Gee. 2008. "Symmetric Diffeomorphic Image Registration with Cross-Correlation: Evaluating Automated Labeling of Elderly and Neurodegenerative Brain." Medical Image Analysis 12 (1): 26–41. https://doi.org/10.1016/j.media.2007.06.004.

Behzadi, Yashar, Khaled Restom, Joy Liau, and Thomas T. Liu. 2007. "A Component Based Noise Correction Method (CompCor) for BOLD and Perfusion Based fMRI." NeuroImage 37 (1): 90–101. https://doi.org/10.1016/j.neuroimage.2007.04.042.

Ciric, R., William H. Thompson, R. Lorenz, M. Goncalves, E. MacNicol, C. J. Markiewicz, Y. O. Halchenko, et al. 2022. "TemplateFlow: FAIR-Sharing of Multi-Scale, Multi-Species Brain Models." Nature Methods 19:

1568–71. https://doi.org/10.1038/s41592-022-01681-2.

Cox, Robert W., and James S. Hyde. 1997. "Software Tools for Analysis and Visualization of fMRI Data." NMR in Biomedicine 10 (4-5): 171–78. https://doi.org/10.1002/(SICI)1099-1492(199706/08)10:4/5<171::AID-NBM453>3.0.CO;2-L.

Dale, Anders M., Bruce Fischl, and Martin I. Sereno. 1999. "Cortical Surface-Based Analysis: I. Segmentation and Surface Reconstruction." NeuroImage 9 (2): 179–94. https://doi.org/10.1006/nimg.1998.0395.

Esteban, Oscar, Ross Blair, Christopher J. Markiewicz, Shoshana L. Berleant, Craig Moodie, Feilong Ma, Ayse Ilkay Isik, et al. 2018. "fMRIPrep 23.0.2." Software. https://doi.org/10.5281/zenodo.852659.

Esteban, Oscar, Christopher Markiewicz, Ross W Blair, Craig Moodie, Ayse Ilkay Isik, Asier Erramuzpe Aliaga, James Kent, et al. 2019. "fMRIPrep: A Robust Preprocessing Pipeline for Functional MRI." Nature Methods 16: 111–16. https://doi.org/10.1038/s41592-018-0235-4.

Evans, AC, AL Janke, DL Collins, and S Baillet. 2012. "Brain Templates and Atlases." NeuroImage 62 (2): 911–22. https://doi.org/10.1016/j.neuroimage.2012.01.024.

Fonov, VS, AC Evans, RC McKinstry, CR Almli, and DL Collins. 2009. "Unbiased Nonlinear Average Age-Appropriate Brain Templates from Birth to Adulthood." NeuroImage 47, Supplement 1: S102. https://doi.org/10.1016/S1053-8119(09)70884-5.

Gorgolewski, K., C. D. Burns, C. Madison, D. Clark, Y. O. Halchenko, M. L. Waskom, and S. Ghosh. 2011. "Nipype: A Flexible, Lightweight and Extensible Neuroimaging Data Processing Framework in Python." Frontiers in Neuroinformatics 5: 13. https://doi.org/10.3389/fninf.2011.00013.

Gorgolewski, Krzysztof J., Oscar Esteban, Christopher J. Markiewicz, Erik Ziegler, David Gage Ellis, Michael Philipp Notter, Dorota Jarecka, et al. 2018. "Nipype." Software. https://doi.org/10.5281/zenodo.596855.

Greve, Douglas N, and Bruce Fischl. 2009. "Accurate and Robust Brain Image Alignment Using Boundary-Based Registration." NeuroImage 48 (1): 63–72. https://doi.org/10.1016/j.neuroimage.2009.06.060.

Jenkinson, Mark, Peter Bannister, Michael Brady, and Stephen Smith. 2002. "Improved Optimization for the Robust and Accurate Linear Registration and Motion Correction of Brain Images." NeuroImage 17 (2): 825–41. https://doi.org/10.1006/nimg.2002.1132.

Klein, Arno, Satrajit S. Ghosh, Forrest S. Bao, Joachim Giard, Yrjö Häme, Eliezer Stavsky, Noah Lee, et al. 2017. "Mindboggling Morphometry of Human Brains." PLOS Computational Biology 13 (2): e1005350. https://doi.org/10.1371/journal.pcbi.1005350.

Lanczos, C. 1964. "Evaluation of Noisy Data." Journal of the Society for Industrial and Applied Mathematics Series B Numerical Analysis 1 (1): 76–85. https://doi.org/10.1137/0701007.

Patriat, Rémi, Richard C. Reynolds, and Rasmus M. Birn. 2017. "An Improved Model of Motion-Related Signal Changes in fMRI." NeuroImage 144, Part A (January): 74–82. https://doi.org/10.1016/j.neuroimage.2016.08.051.

Power, Jonathan D., Anish Mitra, Timothy O. Laumann, Abraham Z. Snyder, Bradley L. Schlaggar, and Steven E. Petersen. 2014. "Methods to Detect, Characterize, and Remove Motion Artifact in Resting State fMRI." NeuroImage 84 (Supplement C): 320–41. https://doi.org/10.1016/j.neuroimage.2013.08.048.

Pruim, Raimon H. R., Maarten Mennes, Daan van Rooij, Alberto Llera, Jan K. Buitelaar, and Christian F. Beckmann. 2015. "ICA-AROMA: A Robust ICA-Based Strategy for Removing Motion Artifacts from fMRI Data." NeuroImage 112 (Supplement C): 267–77. https://doi.org/10.1016/j.neuroimage.2015.02.064.

Reuter, Martin, Herminia Diana Rosas, and Bruce Fischl. 2010. "Highly Accurate Inverse Consistent Registration: A Robust Approach." NeuroImage 53 (4): 1181–96. https://doi.org/10.1016/j.neuroimage.2010.07.020.

Satterthwaite, Theodore D., Mark A. Elliott, Raphael T. Gerraty, Kosha Ruparel, James Loughead, Monica E. Calkins, Simon B. Eickhoff, et al. 2013. "An improved framework for confound regression and filtering for

control of motion artifact in the preprocessing of resting-state functional connectivity data." NeuroImage 64 (1): 240–56. https://doi.org/10.1016/j.neuroimage.2012.08.052.

Tustison, N. J., B. B. Avants, P. A. Cook, Y. Zheng, A. Egan, P. A. Yushkevich, and J. C. Gee. 2010. "N4itk: Improved N3 Bias Correction." IEEE Transactions on Medical Imaging 29 (6): 1310–20. https://doi.org/10.1109/TMI.2010.2046908.

Zhang, Y., M. Brady, and S. Smith. 2001. "Segmentation of Brain MR Images Through a Hidden Markov Random Field Model and the Expectation-Maximization Algorithm." IEEE Transactions on Medical Imaging 20 (1): 45–57. https://doi.org/10.1109/42.906424.

5 FSL analysis

5.1 Regions of interest

We created two masks containing regions of interest, one only containing the bilateral fusiform gyrus, the other additionally containing the following regions: ACC_pre_L, ACC_pre_R, ACC_sub_L, ACC_sub_R, ACC_sup_L, ACC_sup_R, Amygdala_L, Amygdala_R, Insula_L, Insula_R, Precuneus_L, Precuneus_R, SupraMarginal_R and Temporal_Rup_R. All regions were extracted from the AAL3 atlas.

The ROI mask only containing the fusiform gyri was used to assess the colour prediction errors in the comparison group, as well as group differences in neural correlates of colour prediction errors. The other ROI mask was used to evaluate all other hypotheses, including emotion prediction error and prediction strength in the comparison group as well as the pooled sample and group differences in neural correlates of emotion prediction errors.

5.2 Combine FSL output

```
# get one type of input from each contrast
ls.files = dir(pattern = '.*MNI-AAL.csv', path = "./results_sig")

for (file in ls.files) {
  contrast = gsub("_MNI-AAL.csv", "", file)
  type     = substr(contrast, nchar(contrast)-5, nchar(contrast)-5)
  maxima   = read_csv(file.path("results_sig", file), show_col_types = F)
  if (nrow(maxima) == 0) next
  summary  = read_delim(file.path("results_sig",
                                  paste0(contrast, '_cluster-summary.txt')),
                        show_col_types = F)
  output   = read_delim(file.path("results_sig",
                                  paste0(contrast, '_randomise_output_all.txt')),
                        show_col_types = F)

  relinfo  =
    merge(
      output %>% select(`Cluster Index`, Voxels),
      maxima %>% select(`Cluster Index`, `Value`, MNIx, MNIy, MNIz, AALname)
      ) %>%
    mutate(
      H = if_else(MNIx >= 0, "R", "L")
    ) %>%
    rename(
      `Cluster size` = "Voxels",
      "Region" = "AALname",
      "x" = "MNIx",
```

```
      "y" = "MNIy",
      "z" = "MNIz"
    ) %>%
    arrange(desc(`Cluster Index`), desc(Value)) %>%
    relocate(`Cluster Index`, Region, `Cluster size`, H)
  colnames(relinfo)[colnames(relinfo) == "Value"] = paste0(type, "-value")

  write_csv(relinfo, file = file.path("results_sig", paste0(contrast, '.csv')))

}
```

5.3 Hypothesis-guided ROI analysis

```
# COMP: same areas as Stefanics et al. (2019), Neuroimage

read_csv(file.path("results_sig", 'hgf_ctr_eps_c_ROI_fstat1.csv'), show_col_types = F) %>%
  kable(., caption = 'COMP: colour prediction error')
```

Table 7: COMP: colour prediction error

Cluster Index	Region	Cluster size	H	f-value	x	y	z
1	Fusiform gyrus	241	R	24.3	30	-70	-10
1	Fusiform gyrus	241	R	23.1	32	-60	-14
1	Fusiform gyrus	241	R	22.9	28	-60	-16
1	Fusiform gyrus	241	R	20.8	30	-64	-16
1	Fusiform gyrus	241	R	17.8	28	-52	-18
1	Fusiform gyrus	241	R	14.2	32	-78	-6

```
read_csv(file.path("results_sig", 'hgf_ctr_mu_e_ROI_fstat1.csv'), show_col_types = F) %>%
  kable(., caption = 'COMP: emotion prediction strength')
```

Table 8: COMP: emotion prediction strength

Cluster Index	Region	Cluster size	H	f-value	x	y	z
1	Precuneus	3	R	26	6	-76	52

```
# pooled: same areas as Stefanics et al. (2019), Neuroimage

read_csv(file.path("results_sig", 'hgf_all_eps_c_ROI_fstat1.csv'), show_col_types = F) %>%
  kable(., caption = 'Pooled: colour prediction error')
```

Table 9: Pooled: colour prediction error

Cluster Index	Region	Cluster size	H	f-value	x	y	z
7	Fusiform gyrus	230	R	39.8	32	-60	-16
7	Fusiform gyrus	230	R	35.9	28	-70	-12
7	Fusiform gyrus	230	R	24.5	34	-74	-14
6	Superior temporal gyrus	58	R	19.2	50	-40	22
5	Insula	57	R	23.3	32	24	-4

Cluster Index	Region	Cluster size	H	f-value	x	y	z
5	Insula	57	R	22.3	42	26	-4
5	Insula	57	R	16.1	42	22	-10
4	Anterior cingulate cortex, supracallosal	47	R	22.0	8	34	24
3	Fusiform gyrus	28	L	32.1	-28	-56	-16
2	Superior temporal gyrus	15	R	17.9	52	-6	-14
1	Superior temporal gyrus	2	R	13.9	50	-18	-8

```r
read_csv(file.path("results_sig", 'hgf_all_mu_c_ROI_fstat1.csv'), show_col_types = F) %>%
  kable(., caption = 'Pooled: colour prediction strength')
```

Table 10: Pooled: colour prediction strength

Cluster Index	Region	Cluster size	H	f-value	x	y	z
7	Precuneus	2401	L	30.5	-8	-70	40
7	Precuneus	2401	R	28.0	12	-46	42
7	Precuneus	2401	R	27.7	8	-50	42
7	Precuneus	2401	R	26.8	4	-56	50
7	Precuneus	2401	R	25.2	10	-50	46
7	Precuneus	2401	R	24.1	2	-62	48
6	SupraMarginal gyrus	553	R	25.5	58	-44	36
6	SupraMarginal gyrus	553	R	24.4	58	-46	44
6	Superior temporal gyrus	553	R	22.9	48	-40	10
6	SupraMarginal gyrus	553	R	21.9	52	-44	38
6	SupraMarginal gyrus	553	R	21.2	52	-40	44
6	Superior temporal gyrus	553	R	20.4	58	-50	18
5	Anterior cingulate cortex, supracallosal	254	R	22.4	10	24	26
5	Anterior cingulate cortex, pregenual	254	R	20.9	8	40	16
5	Anterior cingulate cortex, pregenual	254	R	15.1	8	46	14
4	Insula	171	L	23.3	-28	22	4
4	Insula	171	L	22.4	-32	20	-8
4	Insula	171	L	21.2	-28	26	-4
4	Insula	171	L	16.8	-36	16	-2
3	Insula	119	R	26.9	32	24	-4
3	Insula	119	R	24.2	38	26	-4
3	Insula	119	R	22.1	42	20	-8
3	Insula	119	R	14.6	44	22	0
2	Precuneus	63	R	13.1	20	-54	20
2	Precuneus	63	R	11.9	14	-54	18
1	Precuneus	3	R	12.5	4	-46	14

```r
read_csv(file.path("results_sig", 'hgf_all_mu_e_ROI_fstat1.csv'), show_col_types = F) %>%
  kable(., caption = 'Pooled: emotion prediction strength')
```

Table 11: Pooled: emotion prediction strength

Cluster Index	Region	Cluster size	H	f-value	x	y	z
4	Precuneus	124	R	23.9	8	-78	56
4	Precuneus	124	R	23.6	6	-74	52
4	Precuneus	124	R	18.2	16	-70	48

Cluster Index	Region	Cluster size	H	f-value	x	y	z
3	Fusiform gyrus	86	R	30.1	32	-52	-20
3	Fusiform gyrus	86	R	21.5	28	-52	-14
3	Fusiform gyrus	86	R	20.4	32	-62	-18
3	Fusiform gyrus	86	R	17.9	30	-62	-14
2	Fusiform gyrus	62	L	24.7	-30	-64	-16
2	Fusiform gyrus	62	L	22.8	-34	-60	-18
2	Fusiform gyrus	62	L	20.4	-34	-54	-18
2	Fusiform gyrus	62	L	17.9	-34	-54	-22
1	Precuneus	2	R	13.2	14	-74	48

```r
# Neural adaptation

read_csv(file.path("results_sig", 'smp_adapt_neg_ROI_tstat1.csv'), show_col_types = F) %>%
  kable(., caption = 'ALL: repetition suppression')
```

Table 12: ALL: repetition suppression

Cluster Index	Region	Cluster size	H	t-value	x	y	z
3	Fusiform gyrus	55	R	4.36	28	-80	-16
3	Fusiform gyrus	55	R	4.24	36	-76	-16
2	Fusiform gyrus	27	R	4.62	38	-56	-22
1	Fusiform gyrus	12	R	4.16	32	-56	-14

5.4 Plotting

Plot the participants' activation in clusters larger than 100 voxels to visualise the effects.

```r
# custom colour palette
custom.col = c("#009E73", "#D55E00", "#0058b2", "#CC79A7")

# load in the extracted activation
df.act = read_csv(file.path("fMRI_data", "grp_use-sorted.csv"),
                  show_col_types = F) %>%
  mutate(
    diagnosis = fct_recode(diagnosis,
                           "COMP" = "CTR")
  ) %>%
  select(diagnosis) %>%
  mutate(
    # load zstats for eps-c
    `colour pwPE-rFG`  = scan(file.path("fMRI_data", "eps_c_C7_meants.txt")),
    `colour pwPE-rSTS` = scan(file.path("fMRI_data", "eps_c_C6_meants.txt")),
    `colour pwPE-rINS` = scan(file.path("fMRI_data", "eps_c_C5_meants.txt")),
    `colour pwPE-rACC` = scan(file.path("fMRI_data", "eps_c_C4_meants.txt")),
    # load zstats for mu-z
    `colour PS-rINS`  = scan(file.path("fMRI_data", "mu_c_C3_meants.txt")),
    `colour PS-lINS`  = scan(file.path("fMRI_data", "mu_c_C4_meants.txt")),
    `colour PS-rACC`  = scan(file.path("fMRI_data", "mu_c_C5_meants.txt")),
    `colour PS-rSMG`  = scan(file.path("fMRI_data", "mu_c_C6_meants.txt")),
    `colour PS-PRC`   = scan(file.path("fMRI_data", "mu_c_C7_meants.txt")),
    # load zstats for mu-e
```

```r
    `emotion PS-rPRC`  = scan(file.path("fMRI_data", "mu_e_C4_meants.txt")),
    `emotion PS-rFG`   = scan(file.path("fMRI_data", "mu_e_C3_meants.txt")),
    `emotion PS-lFG`   = scan(file.path("fMRI_data", "mu_e_C2_meants.txt")),
    # load zstat for neural adaptation
    `RS-rFG`  = scan(file.path("fMRI_data", "adapt_meants.txt"))
  ) %>%
  pivot_longer(cols = -diagnosis, names_to = c("parameter", "region"),
               names_sep = "-", values_to = "activation")

# plot
p = df.act %>%
  ggplot(aes(region, activation, fill = diagnosis, colour = diagnosis)) + #
  geom_rain(rain.side = 'r',
boxplot.args = list(color = "black", outlier.shape = NA, show.legend = FALSE, alpha = .8),
violin.args = list(color = "black", outlier.shape = NA, alpha = .6),
boxplot.args.pos = list(
  position = ggpp::position_dodgenudge(x = 0, width = 0.3), width = 0.3
),
point.args = list(show_guide = FALSE, alpha = .5, size = 0.5),
violin.args.pos = list(
  width = 0.6, position = position_nudge(x = 0.16)),
point.args.pos = list(position = ggpp::position_dodgenudge(x = -0.25, width = 0.1))) +
  scale_fill_manual(values = custom.col) +
  scale_color_manual(values = custom.col) +
  labs(title = "Neural correlates", x = "", y = "z-stat") +
  facet_grid(. ~ parameter, scales = "free", space = "free") +
  geom_hline(yintercept = 0) +
  theme_bw() +
  theme(legend.position = "bottom",
        plot.title = element_text(hjust = 0.5),
        legend.direction = "horizontal",
        legend.title = element_blank(),
        text = element_text(size = 20, family = "Helvetica"),
        legend.box.margin = margin(-20,0, 0, 0)
        )
```

```
## Warning in (function (mapping = NULL, data = NULL, stat = "half_ydensity", :
## Ignoring unknown parameters: `outlier.shape`
```

```r
ggsave("neural_zstat.tiff", plot = p,
       units  = "mm", width  = 270, height = 100, dpi    = 300)
```

```r
# check normal distribution
df.act %>% group_by(diagnosis, parameter, region) %>%
  shapiro_test(activation) %>%
  arrange(region, parameter)
```

```
## # A tibble: 39 x 6
##    diagnosis parameter  region variable   statistic      p
##    <fct>     <chr>      <chr>  <chr>          <dbl>  <dbl>
##  1 ADHD      colour PS  PRC    activation     0.970 0.699
##  2 ASD       colour PS  PRC    activation     0.924 0.0915
##  3 COMP      colour PS  PRC    activation     0.974 0.811
##  4 ADHD      emotion PS lFG    activation     0.920 0.0656
##  5 ASD       emotion PS lFG    activation     0.975 0.821
```

```
## 6 COMP emotion PS lFG activation 0.963 0.545
## 7 ADHD colour PS lINS activation 0.961 0.488
## 8 ASD colour PS lINS activation 0.965 0.594
## 9 COMP colour PS lINS activation 0.979 0.901
## 10 ADHD colour PS rACC activation 0.906 0.0344
## # i 29 more rows
```

```r
# not: colour PE - rACC, rINS, but most are
df.act = df.act %>%
  mutate(
    param_region = paste0(parameter, '_', region)
  )

# compute bayes factor using anovas
bf.log = c()
comb   = c()
for (c in unique(df.act$param_region)) {
  df.small = df.act %>% filter(param_region == c)
  aov = anovaBF(activation ~ diagnosis, data = df.small)
  comb   = c(comb, c)
  bf.log = c(bf.log, aov@bayesFactor$bf)
}

# apply multiple comparison correction after Westfall (1997)
# as described by de Jong (2019)
m = 6 # output of ceil(max(roots([1, -1, -28]))) in MATLAB
c = 0.5^(2/m);
podds = exp(bf.log) * ((1-c)/c)
bf.cor = c()
for (i in 1:length(bf.log)) {
  ph1    = podds[i]/(1 + podds[i])
  bf.cor = c(bf.cor, log(ph1/(1 - ph1)))
}

# put everything into a dataframe
df.aov = data.frame(comb, bf.log, bf.cor) %>%
  separate(comb, into = c("parameter", "region"), sep = "_") %>%
  mutate(
    interpretation = interpret_bf(bf.cor, log = T)
  )

kable(df.aov %>% arrange(bf.cor))
```

parameter	region	bf.log	bf.cor	interpretation
colour PS	rACC	-1.9853210	-3.3326983	strong evidence against
colour pwPE	rINS	-1.8599635	-3.2073408	strong evidence against
colour PS	rINS	-1.8258148	-3.1731921	strong evidence against
colour PS	lINS	-1.5348511	-2.8822284	strong evidence against
RS	rFG	-1.5018543	-2.8492316	strong evidence against
colour PS	rSMG	-1.2851827	-2.6325601	strong evidence against
colour pwPE	rSTS	-1.2690302	-2.6164076	strong evidence against
colour pwPE	rFG	-1.1409374	-2.4883147	strong evidence against
colour PS	PRC	-0.8845863	-2.2319636	moderate evidence against
colour pwPE	rACC	-0.8614937	-2.2088711	moderate evidence against

parameter	region	bf.log	bf.cor	interpretation
emotion PS	rFG	-0.4132379	-1.7606152	moderate evidence against
emotion PS	lFG	0.3435591	-1.0038183	anecdotal evidence against
emotion PS	rPRC	1.2601849	-0.0871925	anecdotal evidence against

5.5 Focusing on fusiform gyrus

```
# load in the extracted activation
df.fg = read_csv(file.path("fMRI_data", "grp_use-sorted.csv"),
                  show_col_types = F) %>%
  mutate(
    diagnosis = fct_recode(diagnosis,
                           "COMP" = "CTR")
  ) %>%
  select(diagnosis) %>%
  mutate(
    # load zstats for eps-c
    `colour pwPE`   = scan(file.path("fMRI_data", "eps_c_rFG_meants.txt")),
    # load zstats for eps-e
    `emotion pwPE`  = scan(file.path("fMRI_data", "eps_e_rFG_meants.txt")),
    ) %>%
  pivot_longer(cols = -diagnosis, names_to = "parameter", values_to = "activation") %>%
  mutate_if(is.character, as.factor)


# check normal distribution
df.fg %>% group_by(diagnosis, parameter) %>%
  shapiro_test(activation) %>%
  arrange(parameter)
```

```
## # A tibble: 6 x 5
##   diagnosis parameter    variable   statistic     p
##   <fct>     <fct>        <chr>          <dbl> <dbl>
## 1 ADHD      colour pwPE  activation     0.945 0.226
## 2 ASD       colour pwPE  activation     0.972 0.762
## 3 COMP      colour pwPE  activation     0.977 0.863
## 4 ADHD      emotion pwPE activation     0.976 0.823
## 5 ASD       emotion pwPE activation     0.951 0.336
## 6 COMP      emotion pwPE activation     0.963 0.556
```

```
aov.pwPE = anovaBF(activation ~ diagnosis * parameter, data = df.fg)
```

```
## Warning: data coerced from tibble to data frame
```

```
# multiple comparison correction
bf.log = aov.pwPE@bayesFactor$bf
podds = exp(bf.log) * ((1-c)/c)
bf.cor = c()
for (i in 1:length(bf.log)) {
  ph1    = podds[i]/(1 + podds[i])
  bf.cor = c(bf.cor, log(ph1/(1 - ph1)))
}

as.data.frame(aov.pwPE@bayesFactor) %>%
```

```r
  select(bf) %>%
  mutate(
    bf.cor = bf.cor
  ) %>%
  arrange(desc(bf.cor)) %>%
  mutate(
    null.int = interpret_bf(bf.cor, log = T),
    next.int = interpret_bf(bf.cor - lead(bf.cor), log = T)
  )
```

```
##                                                       bf    bf.cor
## parameter                                       8.658146  7.310768
## diagnosis + parameter                           6.869716  5.522339
## diagnosis + parameter + diagnosis:parameter     5.141586  3.794209
## diagnosis                                      -1.885774 -3.233151
##                                                                  null.int
## parameter                                         extreme evidence in favour of
## diagnosis + parameter                             extreme evidence in favour of
## diagnosis + parameter + diagnosis:parameter very strong evidence in favour of
## diagnosis                                               strong evidence against
##                                                                  next.int
## parameter                                   moderate evidence in favour of
## diagnosis + parameter                       moderate evidence in favour of
## diagnosis + parameter + diagnosis:parameter  extreme evidence in favour of
## diagnosis
```

```r
# plot
df.fg %>%
  ggplot(aes(parameter, activation, fill = diagnosis, colour = diagnosis)) + #
  geom_rain(rain.side = 'r',
boxplot.args = list(color = "black", outlier.shape = NA, show.legend = FALSE, alpha = .8),
violin.args = list(color = "black", outlier.shape = NA, alpha = .6),
boxplot.args.pos = list(
  position = ggpp::position_dodgenudge(x = 0, width = 0.3), width = 0.3
),
point.args = list(show_guide = FALSE, alpha = .5, size = 0.5),
violin.args.pos = list(
  width = 0.6, position = position_nudge(x = 0.16)),
point.args.pos = list(position = ggpp::position_dodgenudge(x = -0.25, width = 0.1))) +
  scale_fill_manual(values = custom.col) +
  scale_color_manual(values = custom.col) +
  labs(title = "Right fusiform gyrus", x = "", y = "z-stat") +
  geom_hline(yintercept = 0) +
  theme_bw() +
  theme(legend.position = "bottom",
        plot.title = element_text(hjust = 0.5),
        legend.direction = "horizontal",
        legend.title = element_blank(),
        text = element_text(size = 15, family = "Helvetica")
        )
```

```
## Warning in (function (mapping = NULL, data = NULL, stat = "half_ydensity", :
## Ignoring unknown parameters: `outlier.shape`
```

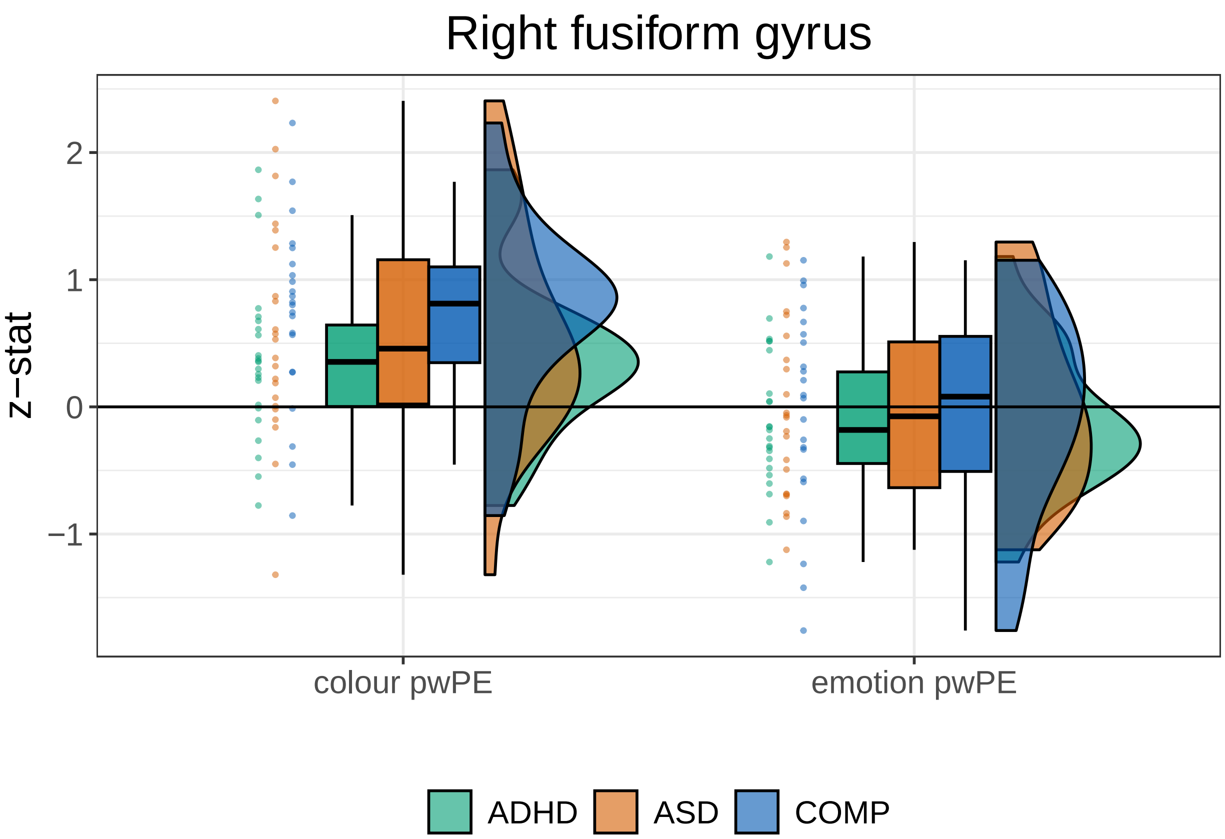


```r
ggsave("rFG_zstat.tiff",
       units  = "mm", width  = 170, height = 100, dpi    = 300)
```
